# Field-Based High-Throughput Plant Phenotyping Reveals the Temporal Patterns of Quantitative Trait Loci Associated with Stress-Responsive Traits in Cotton

**DOI:** 10.1534/g3.115.023515

**Published:** 2016-01-27

**Authors:** Duke Pauli, Pedro Andrade-Sanchez, A. Elizabete Carmo-Silva, Elodie Gazave, Andrew N. French, John Heun, Douglas J. Hunsaker, Alexander E. Lipka, Tim L. Setter, Robert J. Strand, Kelly R. Thorp, Sam Wang, Jeffrey W. White, Michael A. Gore

**Affiliations:** * Plant Breeding and Genetics Section, School of Integrative Plant Science, Cornell University, Ithaca, New York 14853; † Department of Agricultural and Biosystems Engineering, University of Arizona, Maricopa Agricultural Center, Arizona 85138; ‡ Arid-Land Agricultural Research Center, United States Department of Agriculture–Agricultural Research Service (USDA-ARS), Maricopa, Arizona 85138; § Department of Crop Sciences, University of Illinois, Urbana, Illinois 61801; ** Soil and Crop Sciences Section, School of Integrative Plant Science, Cornell University, Ithaca, New York 14853; †† School of Plant Sciences, University of Arizona, Maricopa Agricultural Center, Arizona 85138

**Keywords:** field-based HTPP, stress response, canopy temperature, NDVI, QTL

## Abstract

The application of high-throughput plant phenotyping (HTPP) to continuously study plant populations under relevant growing conditions creates the possibility to more efficiently dissect the genetic basis of dynamic adaptive traits. Toward this end, we employed a field-based HTPP system that deployed sets of sensors to simultaneously measure canopy temperature, reflectance, and height on a cotton (*Gossypium hirsutum* L.) recombinant inbred line mapping population. The evaluation trials were conducted under well-watered and water-limited conditions in a replicated field experiment at a hot, arid location in central Arizona, with trait measurements taken at different times on multiple days across 2010–2012. Canopy temperature, normalized difference vegetation index (NDVI), height, and leaf area index (LAI) displayed moderate-to-high broad-sense heritabilities, as well as varied interactions among genotypes with water regime and time of day. Distinct temporal patterns of quantitative trait loci (QTL) expression were mostly observed for canopy temperature and NDVI, and varied across plant developmental stages. In addition, the strength of correlation between HTPP canopy traits and agronomic traits, such as lint yield, displayed a time-dependent relationship. We also found that the genomic position of some QTL controlling HTPP canopy traits were shared with those of QTL identified for agronomic and physiological traits. This work demonstrates the novel use of a field-based HTPP system to study the genetic basis of stress-adaptive traits in cotton, and these results have the potential to facilitate the development of stress-resilient cotton cultivars.

Cotton (*Gossypium* spp.) is the top renewable textile fiber in the world, supporting a multibillion dollar industry with a global production of 26.2 million metric tons in 2014 (Cotton Inc. 2015). The United States is the third largest producer, with its 2014 crop valued at over $5 billion, generating $25 billion in products and services (USDA-NASS 2015, USDA-ERS 2015). The future sustainability of US cotton production, however, is threatened by climatic changes because nearly 60% of cotton acreage depends on dryland (rainfed) agricultural production systems (National Cotton Council of America 2015). Due to this reliance on precipitation for crop production, the effects of global climate change, including decreased rainfall, increased temperatures, and highly variable weather patterns, pose an imminent risk to cotton production.

Water deficit and high temperature are significant abiotic stresses that often coincide in the production environment, and can dramatically reduce crop yields (Rizhsky *et al.* 2002). In cotton, impacts of high heat, drought, and their combined effects include reproductive limitations through abnormal floral development and fertilization, reduced photosynthetic capacity, impaired photoassimilate distribution, and generation of reactive oxygen species (Burke and Wanjura 2010; Loka *et al.* 2011; Dabbert and Gore 2014). These factors negatively impact lint yield through numerous avenues, including earlier floral cutout, decreased nodes above white flower, reduction in the number of bolls, and impaired carbon assimilation (Chaves *et al.* 2003; Pettigrew 2004). To cope with these environmental challenges, cotton employs mechanisms to regulate water usage and maintain thermal stability, including the use of evaporative cooling through increased stomatal conductance. To investigate the relationship between stomatal conductance and leaf temperature, Radin *et al.* (1994) constructed a Pima cotton (*Gossypium barbadense* L.) population in which these two traits were cosegregating, and evaluated the population in a hot, arid environment. They observed a strong inverse relationship between leaf temperature and stomatal conductance, as well as a significant correlation between cooler canopies and boll set. Such an increase in transpiration rate serves as a mechanism for “heat avoidance,” thereby allowing for the maintenance of plant function.

The development of cultivars possessing tolerance to heat and drought stress is a major consideration of many cotton breeding programs. However, progress has been hampered by a limited understanding of the key genes and alleles that underlie physiological and developmental mechanisms, and how they relate to productivity under abiotic stress, highlighting the current challenge of connecting genotype to phenotype. Despite the substantial evolution of DNA sequencing technologies over the past 10 yr, collection of data for important physiological and developmental phenotypes on large populations remains onerous (Furbank and Tester 2011; Davey *et al.* 2011). In particular, the process of obtaining highly heritable phenotypes associated with tolerance to heat and drought stress is particularly burdensome, given that environmental conditions in which phenotypes were collected are nearly impossible to replicate across field locations and years (Campos *et al.* 2004; Araus and Cairns 2014). The challenges of collecting such data for dynamic traits are further compounded because their observed values are partially dependent upon ambient environmental conditions that could drastically vary within and between days of a single year.

The continued technological advancement of field-based high-throughput plant phenotyping (HTPP) tools has been strongly advocated to address the phenotyping needs of the plant science community (White *et al.* 2012). As one form of proximal sensing, HTPP typically relies on quantifying the interaction of electromagnetic radiation with the plant canopy, using various sensors in close proximity to the plants (Mulla 2013). Due to the noncontact nature of the sensors, and their placement on vehicles capable of traversing research plots at a rapid pace, HTPP systems are capable of collecting vast amounts of data in an efficient manner (Andrade-Sanchez *et al.* 2014; White *et al.* 2012; Busemeyer *et al.* 2013). The ability to collect data rapidly also permits comprehensive assessment of crop development, and, with it, the ability to map QTL expression as a function of time, which is critical given that most traits of agronomic and economic importance are dynamic in nature (Wu and Lin 2006; Würschum *et al.* 2014). Despite awareness of this reality, the majority of QTL studies rely on phenotypic data collected at a single time point, offering only a final view of accumulated QTL effects. By implementing an HTPP system that is capable of collecting data throughout the season under actual production conditions, it becomes possible for researchers to more deeply understand the complexities of trait development and, with this, to better optimize genotypes through selection in breeding programs.

In the present study, we used a high-clearance tractor retrofitted with a suite of sensors to collect data on canopy properties including canopy temperature, reflectance, and height for characterizing a cotton (*Gossypium hirsutum* L.) mapping population of 95 recombinant inbred lines (RILs) under contrasting irrigation regimes. In addition, physiological, agronomic, and fiber quality data were collected so that their genetic relationship with HTPP canopy traits could be investigated. The objectives of this study were to (i) identify QTL responsible for the dynamic response of HTPP canopy traits to the abiotic stresses of high temperature and water deficit; (ii) evaluate the temporal patterns of QTL expression over the reproductive phase of the plant lifecycle; and (iii) examine the genetic relationship of HTPP canopy traits with agronomic, physiological, and fiber quality traits.

## Materials and Methods

### Plant materials and experimental design

The TM-1 × NM24016 mapping population (Gore *et al.* 2012; Percy *et al.* 2006) of 95 RILs was evaluated at the Maricopa Agricultural Center (MAC) of the University of Arizona, located in Maricopa, AZ (33°04’37” N, 111°58’26” W, elevation 358 m) in three consecutive years (2010–2012). The set consisting of repeated parental lines (TM-1 and NM24016), repeated commercial check cultivars (DP 491, FM 958, STV 457, STV 506, and DP 393), and the 95 RILs was evaluated under well-watered (WW) and water-limited (WL) conditions. The experimental field trials were planted on days 127, 117, and 117 (Julian calendar) in 2010, 2011, and 2012, respectively. In each year, the experimental trial was arranged as an 11 × 10 α (0, 1) lattice design with two replications, with a total of 440 plots. The order of entries within each incomplete block was randomized. In addition, the positions of replicates within the experimental field were randomized across years. To reduce edge effects, a conventional commercial upland cotton cultivar was planted on all sides of each replicate. Experimental units were one-row plots, 8.8 m in length, with a 0.61 m alley at the end of each plot. Plots were thinned to a density of ∼4.1 plants m^–2^ and had a spacing between rows of 1.02 m. The soil type is a Casa Grande sandy loam (fine-loamy, mixed, superactive, hyperthermic Typic Natrargids). Conventional cotton cultivation practices for the desert Southwest were employed. Meteorological data were obtained from an automated Arizona Meteorological Network (AZMET) weather station (http://ag.arizona.edu/azmet/index.html) located 270 m from the field (Brown 1989).

Several furrow irrigations were applied during the first 10–14 d after planting to establish the crop, after which subsurface drip irrigation (SDI) was used for the remainder of the field season. The scheduling of SDI was performed using a daily soil water balance model calculated for the cotton root zone as previously described in Andrade-Sanchez *et al.* (2014). Soil water balance model inputs included estimated daily evapotranspiration as determined from FAO-56 crop coefficient procedures (Allen *et al.* 1998), metered irrigation depths, and precipitation data from the AZMET weather station. Soil water characteristics used in the soil water balance were as presented in Table 3 in Hunsaker *et al.* (2005) for sandy loam soil. Irrigations to the WW plots were applied to refill the root zone water content to field capacity at approximately 35% soil water depletion. Starting mid-July, the WL plots received one-half of the irrigation amounts applied to the WW plots. To minimize the interaction of phenology and soil moisture deficit, the WL treatment was imposed when more than 50% of the plots were at first flower. Weekly soil water content measurements in 0.2 m increments from a depth of 0.1 to 1.5 m were made in some plots to monitor the actual soil water depletion and adjust the modeled soil water balance when needed.

### Phenotyping of agronomic, fiber, and physiological traits

The RIL population, parental lines, and commercial check cultivars were phenotyped for a number of agronomic, fiber quality, and physiological traits. The classification of the RIL population for distinct cotton plant developmental stages (flowering/peak bloom; boll development and fill; fiber development and elongation) was based on the number of days after planting and within field plant phenological observations following Oosterhuis (1990). Throughout the growing season, and after mechanical harvest, median plant height for each plot was manually measured with a calibrated bar-coded ruler according to Andrade-Sanchez *et al.* (2014). At the end of the season, plots were mechanically harvested using a one-row harvester. Prior to this, 25 bolls were harvested by hand from each plot and processed using a laboratory 10-saw gin to collect boll and fiber data. The following phenotypic data were collected on the boll and fiber samples: boll size (grams boll^–1^), lint yield (kg ha^–1^), and seeds per boll. The fiber quality traits measured were fiber elongation (percent), strength (kN m kg^–1^), uniformity (percent), micronaire (unit), and length (upper half mean, mm). Fiber quality measurements were made using an Uster HVI 1000 (High Volume Instrument, Uster, Charlotte, NC) at Cotton Incorporated (Cary, NC).

The concentrations of abscisic acid (ABA, picomolar cm^–2^), and soluble sugar (sucrose and glucose, micromolar cm^–2^), were quantified in leaf tissue using an enzyme-linked immunosorbant assay (ELISA) following the method of Setter *et al.* (2010) with additional information provided in Setter *et al.* (2001). Briefly, in 2011 and 2012, leaf tissue samples were taken from six representative plants of each plot, with one leaf disc sample taken per plant. Each leaf disc was collected from the upper lobe of a fully expanded leaf near the third node of the plant. Leaf disc samples were collected on days 237 and 242 (Julian calendar) in 2011 and 2012, respectively, which corresponded to the fiber development and elongation phase of cotton plant development. Leaf discs were taken with a 6-mm punch, and sampled directly into 1.2-ml tubes of a 96-well plate that was promptly stored on ice in a Styrofoam cooler until brought out of the field. Once transferred to the lab, tissue samples were preserved until measuring their concentration of ABA and soluble sugar.

Carbon isotope composition analysis was performed on leaf tissue samples by the University of California, Davis Stable Isotope Facility (Davis, CA). In 2010–2012, leaf disc sample collection was performed using the same protocol as performed for the quantification of ABA and soluble sugar. In 2010, leaf disc samples were collected on day 231 (Julian calendar), which corresponded with the end of cotton boll development and fill. In 2011 and 2012, leaf disc samples were collected on days 251 and 249 (Julian calendar) respectively, which coincided with cotton fiber development and elongation. Dried leaf tissue samples were ground to a fine powder, followed by the weighing and placing of 1–2 mg subsamples into capsules. Carbon isotope composition was determined with an isotope ratio mass spectrometer (Sercon Ltd., Cheshire, UK), and calculated as δ^13^C (‰) relative to the international Vienna Pee Dee Belemnite (V-PDB) reference standard (Farquhar *et al.* (1989). Carbon isotope discrimination (Δ^13^C) was then estimated by the method of Farquhar *et al.* (1989).

### HTPP of canopy traits

We employed an HTPP system to rapidly collect proximally sensed plant canopy temperature, reflectance, and height data from the field experiment over the growing season in 2010–2012. The design, development, operational parameters, and field evaluation of this system have been previously described in Andrade-Sanchez *et al.* (2014). Briefly, a LeeAgra 3434 DL open rider sprayer (LeeAgra, Lubbock, TX) was retrofitted with four sets of three sensor types to simultaneously collect phenotypic data from four adjacent rows of experimental plots (*i.e.*, one set of sensors per row). The three types of sensors used were an Apogee SI-121 infrared radiometer (IRT, Apogee Instruments, Logan, UT) to measure canopy temperature (°C), a CropCircle ACS-470 multi-spectral crop canopy sensor (Holland Scientific, Lincoln, NE) to measure canopy reflectance (ρ) in three 10-nm wavebands with band centers at 670, 720, and 820 nm, and a short-range Pulsar dB3 transducer (Pulsar Process Measurement Ltd., Malvern, UK) to measure canopy height (mm). The wavelength data collected from the CropCircle multi-spectral sensors were used to calculate the normalized difference vegetation index (NDVI) as follows:

$$\mathrm{NDVI}=\left({\text{ρ}}_{\text{ΝΙR}}-{\text{ρ}}_{\text{red}}\right)/\left({\text{ρ}}_{\text{ΝΙR}}+{\text{ρ}}_{\text{red}}\right),$$ (1)

where ρ_NIR_ is the spectral reflectance at wavelength 820 nm in the near-infrared waveband region, and ρ_red_ is the spectral reflectance at wavelength 670 nm in the red waveband region.

To position the HTPP system with centimeter-level accuracy, we used a global navigation satellite system (GNSS) real-time kinematics (RTK) global positioning system (GPS) receiver (A320 Smart Antenna, Hemisphere GPS, Scottsdale, AZ), a rover receiver mounted at the center of the tractor front-mounted frame, and a separate base station unit (A321 Smart Antenna, Hemisphere GPS) to broadcast high-precision positioning information. Raw data generated by the sensors were stored on three data loggers: CR3000 (Campbell Scientific, Logan, UT) for IRT sensors; GeoScout GLS-420 (Holland Scientific, Lincoln, NE) for CropCircle multi-spectral sensors; and CR1000 (Campbell Scientific, Logan, UT) for ultrasonic proximity sensors. The serial output from the GPS receiver was split in order to send identical positioning data to all three data loggers. The semi-automated geospatial postprocessing of the collected data were performed with custom scripts implemented in the open-source Quantum Geographic Information System software (http://www.qgis.org/en/site/) as described in Andrade-Sanchez *et al.* (2014).

In 2010, plant canopy trait data were collected on four different days, while in 2011 and 2012 data were collected on nine and seven different days, respectively. Within each day, canopy temperature, reflectance, and height data were usually collected at multiple times of day, ranging from one to three times in 2010 and 2011, to as many as four times per day in 2012. Canopy height measurements were not collected with the HTPP system in 2010. Measurements were taken in the early morning (0700 or 0900), midmorning (1000 or 1100), afternoon (1300), and/or late afternoon (1500) with all times reported in Mountain Standard Time (MST). The approximate time of day (0700, 0900, 1000, 1100, 1300, or 1500) when data were collected is referred as time of day (TOD), while the actual time, measured in minutes, when a measurement was taken is referred to as time of measurement (TOM). The HTPP system only required ∼0.5 hr to traverse the complete set of experimental plots.

### Statistical analyses

To assess whether the non-HTPP (*i.e.*, agronomic, fiber quality, and physiological traits), and postprocessed HTPP (*i.e.*, plant canopy temperature, NDVI, and height traits) data contained significant outliers, we initially fitted a mixed linear model for each trait with the MIXED procedure in SAS for Windows, version 9.4 (SAS Institute, Cary, NC). When conducting this analysis, an environment was considered as a separate year for non-HTPP traits, and a single day for HTPP traits. For each environment, the fitted model for an individual trait included the main effects of genotype (RILs, parental lines, and commercial check cultivars) and irrigation regime, with their two-way interaction as a fixed effect; replication nested within irrigation regime, and block nested within the two-way interaction of replication and irrigation regime, were included as random effects. Degrees of freedom were calculated via the Satterthwaite approximation. The Studentized deleted residuals (Neter *et al.* 1996) obtained from these mixed linear models were examined to detect outliers. Once significant outliers were removed from the data sets, plot-level averages were calculated with the MEANS procedure in SAS for Windows version 9.4 (SAS Institute).

With the 2011 and 2012 plot-level averages for canopy NDVI, and height traits collected by the HTPP system, the method of Scotford and Miller (2004) was used to calculate a compound canopy index (CCI), from which leaf area index (LAI) was estimated as follows:

$$\mathrm{LAI}=\text{β}\times \mathrm{CCI}=\text{β}\times \left(c/c_{\text{max}}\right)\times \left(h/h_{\text{max}}\right),$$ (2)

where β is a constant, *c* and *h* are the plot-level averages of canopy cover and height, respectively, for each plot, and *c*_max_ and *h*_max_ are, respectively, the maximum values of canopy cover, and height obtained over the growing season. As previously determined from an analysis of height data collected from upland cotton field experiments conducted at MAC from 2009 to 2013, a value of 5.5 was used for β (Thorp *et al.* 2015).The plot-level averages of NDVI were used as estimates of *c*.

For each non-HTPP trait, an iterative mixed linear model fitting procedure was conducted across years in ASReml-R version 3.0 (Gilmour *et al.* 2009):

$$\begin{array}{c} Y_{\mathrm{ijklmn}}=\mu +\mathrm{yea}r_{i}+\mathrm{ir}g_{j}+\mathrm{rep}{\left(\mathrm{irg}\times \mathrm{year}\right)}_{ijk}\\ +\mathrm{column}{\left(\mathrm{rep}\times \mathrm{irg}\times \mathrm{year}\right)}_{ijkl}+\mathrm{block}\;{\left(\mathrm{rep}\times \mathrm{irg}\times \mathrm{year}\right)}_{ijkm}\\ +\mathrm{genotyp}e_{n}+{\left(\mathrm{genotype}\times \mathrm{year}\right)}_{in}+{\left(\mathrm{genotype}\times \mathrm{irg}\right)}_{jn}\\ +\varepsilon_{ijklmn}, \end{array}$$ (3)

in which Y*_ijklmn_* is an individual phenotypic observation; µ is the grand mean; year*_i_* is the effect of the *i*th year; irg*_j_* is the effect of the *j*th irrigation regime (WW or WL); rep(irg × year)*_ijk_* is the effect of the *k*th replication within the *j*th irrigation regime within the *i*th year; column(rep × irg × year)*_ijkl_* is the effect of the *l*th plot grid column within the *k*th replication within the *j*th irrigation regime within the *i*th year; block(rep × irg × year)*_ijkm_* is the effect of the *m*th incomplete block within the *k*th replication within the *j*th irrigation regime within the *i*th year; genotype*_n_* is the effect of the *n*th genotype; (genotype × year)*_in_* is the interaction effect between the *n*th genotype and the *i*th year; (genotype × irg)*_jn_* is the interaction effect between the *n*th genotype and the *j*th irrigation regime; and ε*_ijklmn_* is the random error term following a normal distribution with mean 0 and variance σ*^2^*. The model terms genotype*_n_*, irg*_j_*, and (genotype × irg)*_jn_* were fitted as fixed effects, while all the other terms were fitted as random effects. Likelihood ratio tests were conducted to remove all terms fitted as random effects from the model that were not significant at α = 0.05 (Littell *et al.* 2006).

For each of the four HTPP canopy traits (temperature, height, NDVI, and LAI), an iterative repeated measures mixed linear model fitting procedure was conducted separately for each day in ASReml-R version 3.0 (Gilmour *et al.* 2009):

$$\begin{array}{c} Y_{ijklmo}=\mu +\mathrm{to}{\text{d}}_{i}+\mathrm{ir}{\text{g}}_{j}+{\left(\mathrm{tod}\times \mathrm{irg}\right)}_{ij}\\ +\mathrm{rep}{\left(\mathrm{irg}\times \mathrm{tod}\right)}_{ijk}+\mathrm{column}{\left(\mathrm{rep}\times \mathrm{irg}\times \mathrm{tod}\right)}_{ijkl}\\ +\mathrm{block}{\left(\mathrm{rep}\times \mathrm{irg}\times \mathrm{tod}\right)}_{ijkm}\\ +\mathrm{genotyp}{\text{e}}_{o}+{\left(\mathrm{genotype}\times \mathrm{tod}\right)}_{io}+{\left(\mathrm{genotype}\times \mathrm{irg}\right)}_{jo}\\ +{\left(\mathrm{genotype}\times \mathrm{irg}\times \mathrm{tod}\right)}_{ijo}\\ +\varepsilon_{ijklmno}, \end{array}$$ (4)

with

$$\varepsilon_{ijklmno}\mathrm{equal}\;\mathrm{to}\;Var\left(\varepsilon_{ijklmno}\right)=\sigma^{2},Cov\left(\varepsilon_{ijklmno,}\varepsilon_{i^{\prime }jklmno}\right)=\rho \;\sigma^{2},i\neq i^{\prime }$$

in which Y*_ijklmo_* is an individual plot-level average; µ is the grand mean; tod*_i_* is the effect of the *i*th time of measurement within a day; irg*_j_* is the effect of the *j*th irrigation regime; (tod × trt)*_ij_* is the effect of the interaction between the *i*th time of measurement within a day and the *j*th irrigation regime; rep(irg × tod)*_ijk_* is the effect of the *k*th replication within the *j*th irrigation regime within the *i*th time of measurement within a day; column(rep × irg × tod)*_ijkl_* is the effect of the *l*th plot grid column within the *k*th replication within the *j*th irrigation regime within the *i*th time of measurement within a day; block(rep × irg × tod)*_ijkm_* is the effect of the *m*th incomplete block within the *k*th replication within the *j*th irrigation regime within the *i*th time of measurement within a day; tom(irg × tod)*_ijn_* is the effect of the *n*th minute the measurement was taken within the *j*th irrigation treatment within the *i*th time of measurement within a day; genotype*_o_* is the effect of the *o*th genotype; (genotype × tod)*_io_* is the effect of the interaction between the *o*th genotype and the *i*th time of measurement within a day; (genotype × irg)*_jo_* is the effect of the interaction between the *o*th genotype and the *j*th irrigation regime; (genotype × irg × tod)*_ijo_* is the effect of the interaction between the *o*th genotype, the *j*th irrigation regime, and the *i*th time of measurement within a day; and ε*_ijklmno_* is the random error term following a normal distribution with mean 0 and variance σ*^2^*. The residual variance, ε*_ijklmno_*, was modeled using a correlated error variance structure that incorporated a constant, nonzero, correlation term (ρ) among error terms to account for correlation among multiple measures on the same experimental unit. The following terms were fitted as fixed effects in the model: tod*_i_*; genotype*_o_*; irg*_j_*; (genotype × irg)*_jo_*; (genotype × tod)*_io_*; (tod × irg)*_ij_*; and (genotype × irg × tod)*_ijo_*. All of the other terms were fitted as random effects. Likelihood ratio tests were conducted to remove all terms fitted as random effects from the model that were not significant at α = 0.05 (Littell *et al.* 2006).

The next step of the analysis for each of the non-HTPP and HTPP traits was to detect any remaining influential outliers from the final fitted model on the basis of the DFFITS criterion (Belsley *et al.* 2004; Neter *et al.* 1996) in ASReml-R version 3.0 (Gilmour *et al.* 2009). Once influential observations were removed, the final model (3 or 4) for each trait was refitted to estimate a best linear unbiased estimator (BLUE) for each genotype across years (non-HTPP traits) or within a day (HTPP traits) for the separate irrigation regimes. Sequential tests of fixed effects were conducted, with degrees of freedom being calculated with the Kenward and Rogers approximation (Kenward and Roger 1997) in ASReml-R version 3.0 (Gilmour *et al.* 2009).

For each trait, broad-sense heritability on an entry-mean basis (*Ĥ^2^*) was estimated for the separate irrigation regimes using a mixed linear model. Models (3) and (4) were reformulated to remove the irrigation regime term. Next, all terms were then fitted as random effects in order to obtain variance component estimates. The variance component estimates from each final model for a non-HTPP trait were used to estimate *Ĥ*^2^ (Holland *et al.* 2003) as follows:

$${\hat{H}}^{\text{2}}=\frac{\hat{{\text{σ}}_{g}^{2}}}{\hat{{\text{σ}}_{g}^{2}}+\frac{\hat{{\text{σ}}_{gy}^{2}}}{n_{y}}+\frac{\hat{{\text{σ}}_{\text{ε}}^{2}}}{n_{p}}}=\frac{\hat{{\text{σ}}_{g}^{2}}}{\hat{{\text{σ}}_{p}^{2}}},$$ (5)

where *$\hat{{\text{σ}}_{g}^{2}}$* is the estimated genetic variance, *$\hat{{\text{σ}}_{gy}^{2}}$* is the estimated variance associated with genotype-by-year variation, $\hat{{\text{σ}}_{\text{ε}}^{\text{2}}}$ is the residual error variance, *n_y_* is the harmonic mean of the number of years in which each genotype was observed, and *n_p_* is the harmonic mean of the number of plots in which each genotype was observed. The denominator of equation 5 is equivalent to the phenotypic variance, $\hat{{\text{σ}}_{p}^{2}}$. The variance component estimates from each final model for a HTPP trait were used to estimate *Ĥ*^2^ (Holland *et al.* 2003) as follows:

$${\hat{H}}^{\text{2}}=\frac{\hat{{\text{σ}}_{g}^{2}}}{\hat{{\text{σ}}_{g}^{2}}+\frac{\hat{{\text{σ}}_{\text{ε}}^{2}}}{n_{p}}}=\frac{\hat{{\text{σ}}_{g}^{2}}}{\hat{{\text{σ}}_{p}^{2}}},$$ (6)

where all terms are as previously defined.

Within an irrigation regime, the Pearson’s correlation coefficient (*r*) was used to estimate the degree of association between BLUEs for each pair of traits at α = 0.05 using the *Hmisc* package implemented in R (R Core Team 2014). To allow for the pairwise comparison of non-HTPP *vs.* HTPP traits within a year, BLUEs for non-HTPP traits were estimated for each year with a reformulation of model (3) that excluded the year term. The Bonferroni correction was used to control for the multiple testing problem at α = 0.05/k, with k equal to the number of comparisons.

### QTL analysis

The marker genotyping of, and genetic linkage map construction for, the TM-1 × NM24016 mapping population have been previously described in Gore *et al.* (2014). Briefly, the linkage map consisted of 429 simple-sequence repeat (SSR) and 412 genotyping-by-sequencing (GBS)-based single-nucleotide polymorphism (SNP) marker loci, covering about 50% of the tetraploid cotton genome. These 841 marker loci were assigned to 117 linkage groups, with the number of markers included in each linkage group ranging from 2 to 57. The linkage map covered a total of ∼2061 cM of the tetraploid cotton genome, and the 841 loci were not equally distributed across all 26 chromosomes.

The BLUEs of each non-HTPP trait were used separately to map additive QTL effects in each irrigation regime with inclusive composite interval mapping (ICIM) (Li *et al.* 2007) for biparental populations (BIP) in the QTL IciMapping v. 4.0 software (https://www.integratedbreeding.net). The two stages of the ICIM method have been previously described in Gore *et al.* (2014). In the first stage, the thresholds for individual markers to enter and exit the general linear model via a stepwise regression procedure was set at *P* = 1 × 10^−4^ and *P* = 2 × 10^−4^, respectively, for an overall Type I error rate of α = 0.05 based on a permutation procedure run 1000 times (Anderson and Braak 2003). In the second stage, we conducted a one-dimensional scan across the entire genome at 1-cM steps, based on coefficient estimation in the first stage. In order to select the logarithm of the odds (LOD) threshold for an experiment-wise Type I error rate of α = 0.05, a permutation procedure was run 1000 times (Churchill and Doerge 1994) for each trait in the QTL IciMapping v4.0 software. The LOD thresholds had an average LOD value of 3.3.

We used the BLUEs of each HTPP trait to separately map additive QTL effects and their interaction with the environment (QTL-by-environment interaction, QEI) for each irrigation regime with ICIM for multi-environment trials (MET) (Li *et al.* 2015) in the QTL IciMapping v. 4.0 software. Each day within a year was analyzed independently with time points within a day each considered an environment. Therefore, QTL were mapped across multiple times within a day for an irrigation regime. The two stages of the ICIM MET method are similar to that of ICIM BIP. In the first stage, a permutation procedure was run 1000 times (Anderson and Braak 2003) to set an overall Type I error rate at α = 0.05, resulting in an entry threshold of *P* = 1 × 10^−4^ and exit threshold of *P* = 2 × 10^−4^ that were used to fit individual markers by stepwise regression in a general linear model. In the second stage, for each trait, a one-dimensional scan was carried out across the entire genome at 1-cM steps at an experiment-wise Type I error rate of α = 0.05 (average LOD value of 3.3) as determined by a permutation procedure run 1000 times (Churchill and Doerge 1994) in the QTL IciMapping v4.0 software. The criterion used to declare coincident QTL between traits was based on at least a 10 cM overlap in QTL intervals on the linkage map.

To localize markers on the allotetraploid cotton (*G. hirsutum* L. acc. TM-1) draft genome sequence, we downloaded sequence information for the 26 pseudochromosomes (NBI assembly v1.1; http://www.cottongen.org) (Zhang *et al.* 2015). The BLASTN algorithm in the BLAST+ version 2.2.29 package (stand-alone) (Camacho *et al.* 2009) was used to align context nucleotide sequences of SNP and SSR markers to the reference genome sequence. These BLASTN results were used to tentatively assign the 117 linkage groups to the 26 pseudochromosomes, and approximate the physical proximity of mapped markers defining QTL intervals to annotated genes in the TM-1 draft genome sequence. To assign linkage groups, we relied on majority agreement among markers within a linkage group (*e.g.*, if 20 of 25 markers from a linkage group were placed on A01, and the remaining five markers were placed on D01, that linkage group was assigned to A01). For cases when agreement among markers was ambiguous (only occurred for relatively smaller linkage groups), we also considered marker sequence length, giving preference to those markers with longer context sequence lengths and higher percent matching to the TM-1 draft genome. The values for the percent identity match between marker sequences and the TM-1 draft genome ranged from a minimum of 94.4% to a maximum of 99.9%, with an average of 98.5%. The NBI_Gossypium_hirsutum_v1.1.gene.gff3 annotation file (http://www.cottongen.org) was used to extract candidate gene information.

### Data availability

BLUEs from the fitted linear mixed models for both HTPP and non-HTPP traits are contained in Supporting Information, File S1. Accompanying genotypic data for the 95 RILs are contained in File S2 with accompanying linkage map information. File S3 contains linkage map information for the TM-1 × NM24016 population integrated with the published TM-1 draft genome sequence.

## Results

### Meteorological conditions at the experimental field site

The summer meteorological conditions of the experimental field site in the low desert of central Arizona served as an optimal environment for assessing the effects of heat and drought stress on the levels of phenotypic variation in the TM-1 × NM24016 cotton recombinant inbred mapping population during the 2010–2012 field seasons. However, it was not possible to impose drought stress in the absence of high temperature, as this would require a cooler, arid environment, such as at a higher elevation in Arizona. The average air temperature during daytime hours (0700–1600 hours MST) was 35° from June (early reproductive) to September (boll and fiber maturation)—the time period of the growing season in which the HTPP system was field deployed. Most days had high temperature extremes above 32° (Figure S1), a threshold temperature above which lint yields sharply decrease (Schlenker and Roberts 2009). Among the three field seasons, there were statistically significant differences (*P* < 0.05) for air temperature (°C), relative humidity (%), vapor pressure deficit (kPa), precipitation (mm), and evapotranspiration (mm), but not for solar radiation (MJ/m^2^) (Table S1). The results from analyzing meteorological data from specific days on which measurements were taken by the HTPP system (Table S2) revealed that relative humidity, air temperature, and vapor pressure deficit were significantly different (*P* < 0.01) (Table S1). With the exception of monsoon rainfall events on days 228 and 234 (Julian calendar) of the 2012 field season, precipitation was minimal. These two events led to saturated soil conditions that prevented the use of the HTPP system for these 2 wk. Although years differed for most environmental parameters, the hot, arid environment of central Arizona consistently provided conditions conducive to imposing heat and drought stress on the TM-1 × NM24016 RIL population.

### HTPP of canopy traits

**Figure 1:**
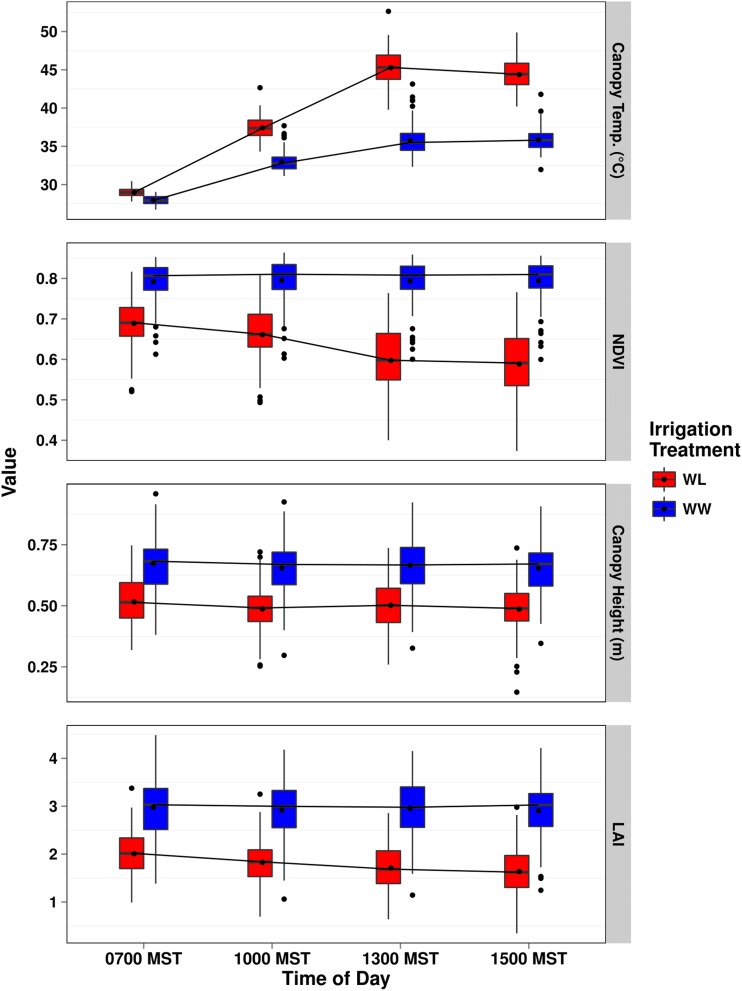
Comparison of best linear unbiased estimators (BLUEs) for canopy temperature (CT, degrees Celsius, top panel), normalized difference vegetation index (NDVI, unitless, second from top), canopy height (meters, third from top), and leaf area index (LAI, unitless, bottom panel) for the two irrigation regimes, water-limited (WL) and well-watered (WW), at four time points (0700, 1000, 1300, and 1500 MST) on day 222 (Julian calendar) in 2012.

We used the HTPP system to evaluate how four plant canopy phenotypes of the TM-1 × NM24016 RIL population responded to high temperature and water deficit under two managed irrigation regimes, WW and WL, at different times of day, from the early reproductive through boll and fiber maturation phases in 2010–2012. The multiple times throughout the day in which measurements of canopy temperature, NDVI, height, and LAI were taken by the HTPP system, or calculated from postprocessed data related to early morning (0700 or 0900), midmorning (1000 or 1100), afternoon (1300), and/or late afternoon (1500) within a maximum of 20 d. Indicative of an elicited physiological response, WL plots exhibited a warmer average canopy temperature compared to WW plots across the three years (Figure S2), with irrigation regime significant (*P* < 0.05 to 0.0001) for canopy temperature for 16 of the 19 d (Table S3). In addition, time of day (*P* < 0.05 to 0.0001) was significant for 18 of 19 d (Table S3). Illustrative of the rapid response that canopy temperature has to stomatal closure under limiting soil moisture, high air temperature, and vapor pressure deficit, early morning measurements (0700 MST) of canopy temperature were often similar between irrigation regimes, but, as time progressed during the day, there was a more rapid change in canopy temperature for WL plots relative to WW plots (Figure 1, Figure S3, Figure S4, Figure S5, Table S4, Table S5, and Table S6).

The WW plots had higher NDVI than WL plots (Figure S6), with the effect of irrigation regime being significant (*P* < 0.05 to 0.0001) for 15 out of 20 d across the three years (Table S7). In contrast to canopy temperature, time of day was significant only 5 of 20 d for NDVI―a trait that is less temporally responsive to environmental stress (Table S7). Inverse to canopy temperature, we found that NDVI for WL plots generally decreased over the course of a day, whereas NDVI for WW plots either remained constant or increased slightly (Figure 1, Figure S7, Figure S8, Figure S9, Table S8, Table S9, and Table S10). Similar to what was observed for Pima cotton (Andrade-Sanchez *et al.* 2014), this change in NDVI for WL plots was due to leaf wilting from loss in turgidity, causing changes to the canopy geometry that resulted in lower reflective values due to greater soil reflectivity in the instrument’s field of view. It is likely that the slight increase in NDVI for WW plots arose from a phenomenon known as diaheliotropism, in which leaves track the movement of the sun (Lang 1973; Ehleringer and Hammond 1987).

The height of the canopy for the TM-1 × NM24016 RIL population from 2011 and 2012 (measured on a total of 14 d) displayed the expected pattern of WW plots having taller canopies than the WL plots (Figure 1 and Figure S10), and a change in height representing incremental plant growth over the season (Figure S11, Figure S12, Table S11, and Table S12). Irrigation regime effects for 2012 followed an anticipated trend in which early season measurements were either not significant or only slightly significant (*P* < 0.05, Table S13) until midseason, when they were all highly significant (*P* < 0.0001). In contrast, treatment effects for 2011 were more variable, in that some early season measurements were highly significant (day 202, *P* < 0.0001), followed by a day where the difference between irrigation regimes was not significant (day 216). This variability likely resulted from clogged irrigation tape and emitters, which prevented uniform water distribution across plots. In order to reestablish experimental field conditions, equal furrow irrigation was applied, which attenuated the height differences between irrigation regimes, but WW plots were still taller than WL plots. In general, time of day was not significant; however, time of day was significant toward the end of the 2012 season (days 222–258) due to either severe wilting, or the weight of bolls, causing less turgid plants in WL plots to lodge.

To gain further insight into the whole canopy response (Gutierrez *et al.* 2012; Thorp *et al.* 2015), we estimated LAI based on canopy NDVI and height from 2011 and 2012. As expected, the WW plots had higher LAI than WL plots in both years (Figure S13). Additionally, LAI exhibited a curvilinear trend across the growing season, reflecting progressive plant development and biomass accumulation (Figure S14 and Figure S15). Time of day was not significant for 12 of 14 d, whereas the effect of irrigation regime was significant (*P* < 0.05 to 0.0001) for 13 of 14 d (Table S14). Consistent with canopy NDVI, WW plots were able to maintain higher values of LAI over the course of a day. However, WL plots experienced a gradual decrease in LAI, which was likely from a continuing increase in leaf wilting that concomitantly lowered NDVI (Figure 1, Table S15, and Table S16).

In general, all HTPP canopy traits were highly heritable (average *Ĥ*^2^ of 0.87) under both irrigation regimes across the three years (Figure S16, Figure S17, Figure S18, Figure S19, Table S17, Table S18, Table S19, and Table S20). The average of broad-sense heritability estimates for both irrigation regimes across years for canopy temperature, NDVI, canopy height, and LAI were 0.80, 0.86, 0.92, and 0.93, respectively, with the highest estimate (*Ĥ*^2^ = 0.98) calculated for NDVI under WL conditions in 2011 and 2012. However, there were days in which estimates were low and nearly zero in one instance (*Ĥ*^2^ = ∼0 for NDVI under both WW and WL, day 188, 2011). The days on which the HTPP traits had relatively lower estimates of heritability typically occurred earlier in the season prior to canopy closure when exposed soil likely influenced the proximally sensed measurements. In addition, the increased environmental variance in heritability estimates for canopy temperature in 2011 (Figure S16) under WW conditions was likely due to the eventually resolved irrigation issues encountered with clogged drip irrigation tape and emitters.

### Agronomic, fiber, and physiological traits

Lint yield, boll size, and seed per boll all exhibited large genotypic and irrigation regime effects (*P* < 0.0001) when assessed over the three growing seasons (Table S21). As expected for complex traits, lint yield displayed a significant genotype-by-irrigation regime interaction (*P* < 0.001) as did seed per boll (*P* < 0.05), thus highlighting their responsiveness to the water deficit treatment. However, broad-sense heritabilities for these three agronomic traits were high across the three years and two irrigation regimes, with estimates ranging from 0.66 to 0.81 (Table 1). Similar to canopy height, the irrigation regime effect for plant height, which was manually measured multiple times throughout each field season, was generally found to be significant (*P* < 0.05 to < 0.0001; Table S22), with WW plots being taller than WL plots (Figure S20, Figure S21, Figure S22, Figure S23, Table S23, Table S24, and Table S25). The range of values for plant height was ∼30% larger than for canopy height. Estimates of broad-sense heritability for plant height on different days ranged from 0.48 to 0.89 for WW and WL conditions (Figure S24 and Table S26).

**Table 1:** Mean, SD, and range of BLUEs for traits evaluated for the TM-1 × NM24106 RIL population evaluated under two irrigation regimes, WL and WW, including parental lines and their midparent values

Trait	Irrigation Regime	Parents			RIL Population				Heritability
		TM-1	NM24016	Midparent	Mean	SD	Minimum	Maximum	*Ĥ*^2^
ABA concentration (pmol cm^–2^)	WL	10.71	8.78	9.75	10.60	2.72	3.52	21.00	0.15
	WW	6.99	6.76	6.88	7.00	1.89	3.18	11.39	0.23
CID (Δ^13^C)	WL	20.28	19.72	20.00	20.25	0.28	19.59	20.80	0.31
	WW	20.50	20.13	20.32	20.49	0.31	19.47	21.14	0.30
Sugar concentration (μmol cm^–2^)	WL	0.43	0.45	0.44	0.47	0.07	0.32	0.62	0.12
	WW	0.40	0.41	0.41	0.41	0.07	0.27	0.71	0.19
Fiber elongation (%)	WL	5.12	4.34	4.73	5.06	0.76	3.19	6.78	0.88
	WW	5.26	4.27	4.77	5.10	0.77	3.26	6.92	0.87
Fiber micronaire (unit)	WL	5.09	4.30	4.70	4.36	0.57	3.19	5.75	0.83
	WW	5.04	4.38	4.71	4.37	0.56	3.09	5.73	0.80
Fiber uniformity (LUI)	WL	82.69	84.17	83.43	82.81	0.94	80.11	84.90	0.88
	WW	83.18	84.64	83.91	82.97	0.98	80.49	84.97	0.87
Fiber strength (kN m kg^–1^)	WL	292.64	326.48	309.61	322.45	23.14	277.63	371.49	0.83
	WW	290.09	327.16	308.63	320.49	21.67	276.46	391.59	0.79
Upper half mean (mm)	WL	1.11	1.18	1.15	1.13	0.05	0.99	1.25	0.81
	WW	1.13	1.19	1.16	1.14	0.06	0.99	1.27	0.83
Boll size (g boll^–1^)	WL	4.73	2.50	3.62	3.29	0.56	2.15	4.66	0.70
	WW	4.98	2.75	3.87	3.51	0.63	2.26	5.26	0.81
Lint yield (kg ha^–1^)	WL	667.25	325.00	496.13	402.61	135.15	178.33	920.16	0.71
	WW	890.60	454.48	672.54	523.55	173.80	138.34	1066.54	0.68
Seed per boll	WL	25.09	13.77	19.43	17.58	2.74	11.43	24.71	0.66
	WW	26.40	14.84	20.62	18.66	2.76	12.50	25.23	0.78

Estimates of broad-sense heritability (*Ĥ*^2^) on an entry-mean basis are reported. Field trials were conducted from 2010 to 2012 at the Maricopa Agricultural Center located in Maricopa, AZ. BLUE, best linear unbiased estimators; RIL, recombinant inbred line; WL, water-limited conditions; WW, well-watered conditions; ABA, abscisic acid; CID, carbon isotope discrimination; LUI, length uniformity index.

The five fiber quality traits investigated in this study, fiber elongation, micronaire, strength, length (upper half mean), and uniformity, exhibited extensive phenotypic variation and high heritability across the three years (average *Ĥ*^2^ of 0.84 across both irrigation regimes). Of these five traits, fiber strength under WW conditions had the lowest estimate of broad-sense heritability (*Ĥ*^2^ = 0.79), while fiber elongation and uniformity under WL conditions had the highest estimate (*Ĥ*^2^ = 0.88). With the exception of fiber length (irrigation regime; *P* < 0.0001), which has a known response to water deficit (Allen and Aleman 2011), and micronaire (genotype-by-irrigation regime; *P* < 0.05), none of the fiber traits showed a significant irrigation regime or genotype-by-irrigation regime interaction (Table S21). Taken together, the relatively high heritabilities and lack of environmental perturbation suggest that variation for these fiber traits is mostly under the control of genetic effects.

The three physiological traits measured in this study, leaf ABA concentration, sugar concentration, and carbon isotope discrimination (CID), showed genotypic differences (*P* < 0.05 to < 0.0001, Table S21), but did not vary with irrigation regime (*P* > 0.05). However, when these traits were analyzed within a year, there were often significant differences between irrigation regimes (Table S27). The estimated broad-sense heritabilities for these traits, averaged across irrigation regimes, were 0.19, 0.31, and 0.16 for ABA, CID, and leaf sugar concentration, respectively. The relatively lower broad-sense heritability estimates for these traits, in combination with the variable treatment effects observed for individual years, highlight the sensitivity of these physiological traits to environmental factors.

### Phenotypic correlations

We investigated the degree of relationship between traits by calculating pairwise correlation coefficients (Pearson’s *r*) between all traits under both WL and WW conditions (File S4, an additional set of BLUEs on a yearly basis was generated for the non-HTPP traits). In assessing the strength of correlations between the HTPP and physiological traits, only CID was found to consistently have a significant correlation (*r* = 0.35 to 0.40; *P* < 0.001) with canopy height on five separate days in 2011 under WW conditions at a Bonferroni correction of 5%. With the exception of canopy temperature, the HTPP traits did not significantly covary with most of the fiber traits on any one day across the three years. Fiber elongation was significantly correlated with canopy temperature during boll maturation for 2010 WL, 2011 WW, and 2012 WL treatments, with a maximum correlation of –0.35 (*P* < 0.05) under WL conditions in 2012. Although not significant at a Bonferroni correction of 5%, micronaire tended to have a weak, negative correlation (*r* < 0.30) across years (maximum *r* of –0.31 in 2010) with canopy temperature.

**Figure 2:**
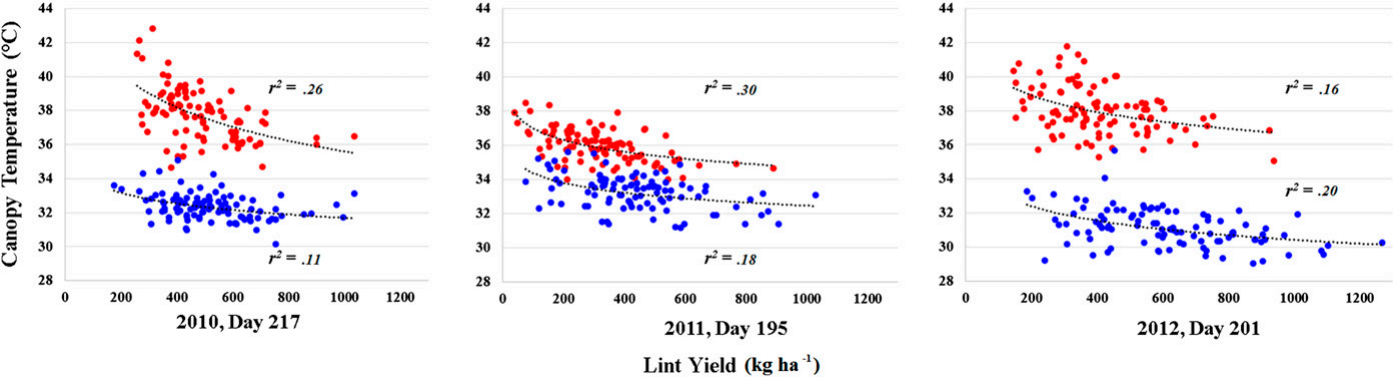
The degree of relationship (multiple *r*^2^ from second order polynomial) between best linear unbiased estimators (BLUEs) of canopy temperature (°C) and lint yield (kg ha^–1^) for the TM-1 × NM24016 recombinant inbred line (RIL) population, and its two parent lines evaluated under two irrigation regimes, water-limited (red) and well-watered (blue). The canopy temperature data were collected from three representative days during flowering/peak bloom within a year (Julian calendar) corresponding to 1300, 1500, and 1300 MST for 2010, 2011, and 2012, respectively.

The correlations of HTPP traits with boll size and lint yield were stronger and more consistent than observed for physiological and fiber quality traits. Significant (*P* < 0.05), negative correlations were found for boll size with canopy temperature (*r* = –0.38 to –0.35), canopy height (*r* = –0.40 to –0.35), and LAI (*r* = –0.40 to –0.35) in both WL and WW irrigation regimes in 2011, although correlations were strongest for the WL regime. The strongest correlations between boll size and canopy temperature were identified early in the season (flowering/peak bloom), whereas boll size was most strongly correlated with canopy height and LAI during the fiber development and elongation phase. We also detected moderately strong correlations for lint yield with canopy temperature and NDVI for both WL and WW plots. Significant correlation values (*P* < 0.05) between lint yield and NDVI ranged from 0.35 to 0.61 for the three years under both irrigation regimes, while for canopy temperature the values ranged from –0.42 to –0.62. When fitting a nonlinear curve to lint yield and canopy temperature for the time point identified with the strongest correlation each year, the fit of the curve (*r*^2^) ranged from 0.11 to 0.30 (Figure 2). In contrast to boll size, the strongest correlations between lint yield and canopy temperature were observed at flowering/peak bloom (Table S28).

**Figure 3:**
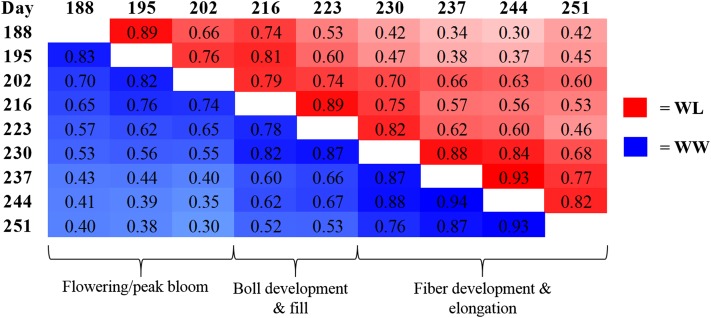
Pearson correlation coefficients (*r*) for best linear unbiased estimators (BLUEs) of canopy temperature, calculated over all time points within a day, under two irrigation regimes, water-limited (red), and well-watered (blue), and their relationship to cotton developmental stages at different days (Julian calendar) throughout the 2011 growing season. All correlations were significant at α = 0.05.

When investigating the degree of relationship between HTPP traits, canopy temperature was found to have significant, negative correlations (*P* < 0.05 to < 0.0001) with NDVI, canopy height, and LAI over all three years for both irrigation regimes. In general, the strongest correlations between canopy temperature and these three canopy HTPP traits was observed in the afternoon at peak physiological stress, irrespective of plant developmental stage. In addition, significant, positive correlations (*P* < 0.05 to < 0.0001) were observed between all pairwise comparisons of canopy height, NDVI, and LAI, but the strength of the association varied across the different developmental phases of plant growth. Interestingly, we found that the strength of the correlations between measurements of a canopy trait within a plant developmental stage was stronger than between stages. This is best exemplified by canopy temperature under both irrigation regimes in 2011, where there is a distinct pattern to the strength of correlations that has a strong relationship with plant phenology (Figure 3 and File S4).

### QTL identification

We mapped QTL controlling phenotypic variation for the HTPP and non-HTPP traits in the TM-1 × NM24016 RIL population. The complete results of QTL mapping results are presented in *Supporting Information* and summarized in Table 2. When conducting the QTL analysis for HTPP traits, each day was analyzed independently, but mapping was performed across multiple time points within a day. Through the implementation of this approach, we generally identified concordant QTL in terms of genomic position associated with HTPP traits across individual days, and multiple years, in both the WL and WW treatments. In total, we identified 14, 15, 14, and 16 QTL for canopy temperature, NDVI, canopy height, and LAI, respectively, at an experiment-wise Type I error rate of α = 0.05, with phenotypic variance explained (PVE) by each QTL ranging from 4.35% to 12.42%.

**Table 2:** Selective summary of QTL identified for HTPP canopy, agronomic, physiological, and fiber quality traits in the TM-1 × NM24016 RIL population under two irrigation regimes, WL and WW, at an experiment-wise Type I error rate of 5%

Chromosome	LG	Left Marker	Left Marker Position (bp)	Left Marker Position (cM)	Right Marker	Right Marker Position (bp)	Right Marker Position (cM)	Interval Size	Traits
A13	55	SNP0144	2,174,006	33.44	SNP0036	4,956,693	54.82	2,782,687	ABA, NDVI (55.00%), LAI (57.14%), CT (36.84%)
A09	32	SNP0003	70,791,307	38.53	SHIN-1542a	74,019,812	4.69	3,228,505	Δ^13^C, CT (31.58%)
A05	74	SNP0029	3,926,888	0.00	SNP0159	5,616,973	24.03	1,690,085	Boll size, CHT (78.57%), NDVI (60.00%), LAI (92.86%), CT (42.11%)
A02	3	SHIN-0129b	40,970,937	11.53	DC40319b	41,271,319	12.81	300,382	Fiber elongation
A11	44	SNP0179	84,855,703	5.32	SNP0140	89,784,339	1.12	4,928,636	Fiber elongation
A11	91	BNL0625a	49,598,610	29.18	BNL2805a	49,637,476	28.68	38,866	Micronaire
D06	112	SNP0361	1,611,037	17.02	SNP0427	1,956,820	13.70	345,783	Micronaire
A06	19	SNP0154	9,290,498	12.87	SNP0070	9,440,198	13.80	149,700	Lint yield, CHT (78.57%)
D01	24	DPL0790a	70,178	0.00	CIR238a	3,374,597	16.60	3,304,419	Lint yield, NDVI (65.00%), CT (73.68%)
D09	99	C2-021a	21,552,954	2.32	SNP0259	21,636,248	0.00	83,294	Lint yield, Seed per boll
A06	21	SNP0426	2,294,776	20.03	SNP0146	2,540,030	9.23	245,254	NDVI (40.00%), LAI (35.71%), CT (36.84%)
A08	29	C2-003a	1,083,826	0.00	SNP0471	1,736,727	16.44	652,901	NDVI (95.00%), LAI (28.57%), CT (63.16%)
D12	54	SNP0331	993,024	0.00	SNP0425	1,432,973	18.45	439,949	CHT (64.29%), NDVI (55.00%), CT (73.68%)
A03	71	SHIN-0690a	2,612,135	0.00	SHIN-0727	2,644,274	10.86	32,140	CHT (57.14%), NDVI (35.00%), LAI (78.57%)
A12	45	MUSB1117a	3,597,960	9.76	SHIN-1413a	10,039,270	33.25	6,441,310	CHT (85.71%), NDVI (65.00%), LAI (85.71%)
D06	22	SNP0086	10,388,600	21.39	SNP0132	23,134,645	0.00	12,746,045	CHT (71.43%), NDVI (60.00%), LAI (85.71%), CT (21.05%)

Marker positions are reported both in centimorgans (cM) on linkage groups and base pairs on respective chromosomes. The complete summary is included in Table S29, Table S30, Table S31, Table S32, Table S33, and Table S34. Values listed in parentheses are the percentage of days on which the QTL were detected. QTL, quantitative trait loci; HTPP, high-throughput plant phenotyping; RIL, recombinant inbred line; WL, water-limited conditions; WW, well-watered conditions; ABA, abscisic acid content; Δ^13^C, carbon isotope discrimination; CHT, canopy height; NDVI, normalized difference vegetation index; LAI, leaf area index; CT, canopy temperature.

For canopy temperature, the 14 QTL were distributed across 13 chromosomes, with one of them detected under WW conditions only (Table S29). Of these 14 QTL, four were detected in all three years under both WL and WW treatments. The total number of days on which these four QTL were identified ranged from six to 14 (canopy temperature was collected on a total of 19 days across the three years). These four QTL were located on the following chromosomes: A08, A09, D10, and D12. The two QTL located on chromosomes D10 and D12 had an average PVE of 6.36% and 8.59%, and a maximum PVE of 13.48% and 14.77% respectively. In addition, the average additive allelic effect estimates of the D10 and D12 QTL were 0.30° and –0.29°, respectively. The QTL on A08 was detected on 12 of the 19 days. This consistent detection was likely due to its relatively large allelic effect estimate, with estimated average and maximum effects of –0.34° (7.59% PVE) and –0.62° (15.17% PVE), respectively. Interestingly, canopy temperature QTL identified on chromosomes A09 and A13 colocalized with QTL identified for leaf CID and ABA concentration, respectively, which were detected under WL conditions. The genomic interval for the CID QTL on chromosome A09 contained a candidate gene (Gene ID: Gh_A13G0355) previously described in *Arabidopsis thaliana* as a member of the *AtDi19* gene family implicated in dehydration and abiotic stress response (Milla *et al.* 2006). In addition, the genomic interval containing the ABA QTL on chromosome A13 included a candidate gene (Gene ID: Gh_A13G0355) identified in *A. thaliana* as an abscisic acid response element (ABRE) binding factor implicated in stress responsive ABA signaling (Kang *et al.* 2002; Kim *et al.* 2004).

The 15 QTL identified for NDVI were located across 14 chromosomes, and, of these, three QTL were associated with only a single irrigation regime (Table S30). Notably, two-thirds of the 15 QTL were shared, based on overlapping genomic positions with those detected for canopy temperature. Given the inverse phenotypic correlation between NDVI and canopy temperature, it was not surprising that the associated allelic effect estimates for the shared QTL were in opposing directions. Of these shared QTL, the QTL identified on chromosome A08 exhibited major control of variation for NDVI. Specifically, this QTL, which had an average PVE of 10.42%, was detected on 19 of the 20 days on which spectral data were collected across the three years.

We identified 14 QTL that controlled variation for canopy height over the three growing seasons (Table S31). As expected, six of these QTL were shared with plant height (Table S32), to which canopy height was also strongly correlated at the phenotypic level (*r* = 0.46 to 0.91, *P* < 0.0001). This result suggests a moderate to high degree of concordance between the two measurement approaches. In addition, the results from QTL mapping of canopy height were comparable to those of LAI, with the genomic positions of 12 QTL shared between the two traits (Table S31 and Table S33). The four QTL not concordant between canopy height and LAI, however, were shared between canopy height and NDVI. These findings were not unexpected given that the calculation of LAI was predominantly based on canopy height and NDVI.

The mapping of QTL for agronomic and fiber quality traits identified a total of 22 QTL that mapped to 11 chromosomes, and of which 18 QTL were unique among traits and irrigation regimes (Table S34). We identified eight QTL for lint yield with allelic effects values ranging from –80.79 (17.85% PVE) to 67.34 kg ha^–1^ (14.76% PVE). Of these QTL, the QTL on D01 mapped to the same genomic location as that of QTL for canopy temperature and NDVI, suggesting at least a partly shared genetic basis between these two HTPP canopy traits and lint yield. Lint yield is very likely to have even more QTL shared with canopy temperature and NDVI, but the small sample size of 95 RILs did not provide sufficient statistical power to identify QTL with small-to-intermediate effects for a polygenic trait such as lint yield (Xu 2003). Regardless, further supporting this association, a boll size QTL on chromosome A05 colocalized with QTL detected for canopy temperature, NDVI, canopy height, and LAI under both irrigation regimes. With respect to the fiber quality traits, none of the identified QTL were shared with those detected for HTPP traits, although we detected only a few unique QTL for fiber elongation, uniformity, and micronaire.

### Patterns of identified QTL

**Figure 4:**
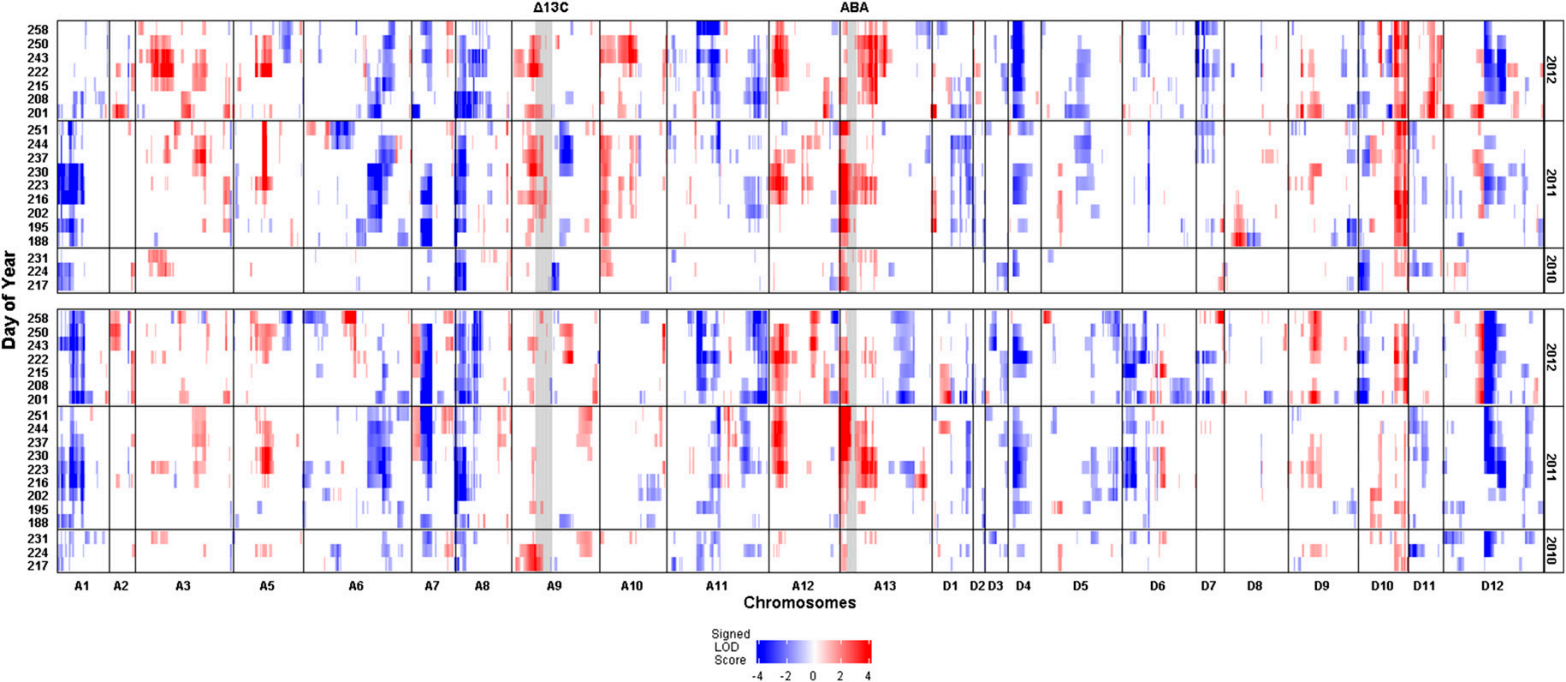
Genome-wide scan for quantitative trait loci (QTL) associated with canopy temperature across three years under two irrigation regimes, water-limited (WL, top panel) and well-watered (WW, bottom panel). Color is representative of the estimated additive allelic effect, with red and blue colors indicating increased and decreased canopy temperature, respectively, when substituting a NM24016 allele with an allele from TM-1. The intensity of the color represents the magnitude of the logarithm of odds (LOD) score with 3.3 as the significance threshold to declare QTL – values above 3.3 were assigned a value of 4 for presentation purposes. Shaded regions represent genomic intervals in which QTL were identified for carbon isotope discrimination and abscisic acid concentration (respective labels above graph). The left-hand y-axis denotes day of the growing season (Julian calendar) that data were collected, and the right-hand y-axis denotes year. The x-axis represents the mapped chromosomes of tetraploid cotton with marker intervals in centimorgans (cM). Chromosomes A04 and D13 did not have any mapped markers.

The HTPP system enabled collection of phenotypic data throughout the season that afforded us the opportunity to assess QTL patterns (*i.e.*, presence *vs.* absence of significant QTL) within and across years, as well as between different irrigation regimes. Of all the HTPP traits, canopy temperature exhibited the most dynamic QTL patterns. Exemplifying this dynamism, QTL identified on chromosomes A09, A12, D04, and D12 showed a strong temporal pattern, varying in frequency of identification from 6 to 14 days (Figure 4 and Table S29). In 2011 and 2012, the canopy temperature QTL on chromosome A12 was not detected until day 216, after which point it was continually detected until approximately day 250 under both irrigation regimes. Similar patterns were also observed for the two canopy QTL on chromosomes D04 and D12, which suggests that all three QTL initiate expression at boll development, and continue throughout the fiber elongation and maturation phase. Finally, the QTL on chromosome A09 was identified predominately under WL conditions at approximately midseason, with its effects diminishing by day ∼250 in 2011 and 2012.

The patterns of identified QTL for NDVI were different from canopy temperature, in that there was less within season variability. The majority of NDVI QTL were either detected for the duration of the season, or beginning midseason at approximately day 220, coinciding with the boll development and fill phase in 2011 and 2012. However, the preferential identification of QTL under a specific irrigation regime was still observed, especially three QTL on chromosomes A10, D06, and D12 (Figure S25). The QTL on chromosome A10, which was detected on eight different days across the three years, displayed the most dramatic specificity as its expression was nonexistent for the WW treatment. The expression of QTL on chromosomes D06 and D12 were exclusively associated to the WW treatment, with the D06 and D12 QTL detected in 2011 and 2012 on 12 and 11 different days, respectively. With respect to QTL patterns for canopy height and LAI, neither trait displayed strong temporal variability, nor irrigation regime specificity as most QTL were repeatedly detected from the beginning of data collection throughout the growing season under both WW and WL conditions (Figure S26 and Figure S27).

## Discussion

The genetic improvement of cotton for yield and fiber quality traits is largely dependent on identifying and understanding key genes and alleles responsible for enhanced productivity under limiting environmental conditions. Central to this challenge is the ability to collect phenotypic data on experimental populations under real-world production conditions encountered throughout the growing season. In that light, we demonstrated the use of an HTPP system to measure canopy traits on a RIL population grown under contrasting irrigation regimes in a hot, arid environment. Through the analysis of data collected by the HTPP system, we mapped QTL controlling the temporal response of canopy traits to heat and drought stress, and demonstrated how these HTPP traits related to agronomic, physiological, and fiber quality traits. To the best of our knowledge, this is the first study using field-based HTPP to investigate the genetic basis of phenotypic responses to heat and drought stress in a genetic mapping population over multiple growing seasons.

Paramount to the success of studying traits that change in response to environmental stimuli is the ability to collect data from experimental plots at multiple time points per day, as this provides a way to quantify the physiological processes underlying dynamic phenotypes. The temporal effects of stomatal closure can be seen by assessing the change in canopy temperature over the course of a day (Figure 1). Day 222 from 2012 was one of the more severe days in terms of high air temperature (maximum temperature of 37.76°), and cooccurring high vapor pressure deficit (VPD, 4.94 kPa). Early in the morning, plant canopy temperatures were approximately equivalent, but, as the day progressed, the two treatments diverged drastically as WW plots were able to maintain lower canopy temperatures, unlike the drought-stressed, WL plots. At peak stress, occurring around 1300 MST, the difference in temperature between the two irrigation regimes was ∼10°, and WL plots had an average canopy temperature of 45°. The elevated canopy temperatures of WL plots is indicative of stomatal closure, mediated through ABA signaling, which is one of the primary plant responses to water deficit, and serves as a mechanism to conserve water resources (Ackerson 1980; Taiz and Zeiger 2006). The closure of stomata leads to elevated canopy temperatures because plants are no longer able to use evaporative cooling, via increased transpiration, to modulate thermal homeostasis (Carmo-Silva *et al.* 2012; Radin *et al.* 1994; Burke and Wanjura 2010). A QTL identified for ABA concentration, which, colocated with a QTL associated with canopy temperature, supports an expected relationship between stomatal conductance and canopy temperature. Additionally, the colocalization of CID and canopy temperature QTL illustrates a shared genetic relationship between these two physiological traits. The positive allelic effects for both QTL imply a reduction in the preferential usage of ^12^C over ^13^C, along with a concomitant increase in canopy temperature, which would result from limited gas exchange between leaf intercellular airspaces and the atmosphere due to stomatal closure (Fischer *et al.* 1998; Farquhar *et al.* 1982).

The high canopy temperatures observed in the WL treatment led to heat stress and its subsequent effects, which likely included reduced flower fertility and boll set, in addition to diminished photosynthetic capacity and eventual overall productivity (Dabbert and Gore 2014; Taiz and Zeiger 2006; Carmo-Silva and Salvucci 2012). The repeatedly observed inverse relationship between canopy temperature and lint yield, an indicator of overall plant productivity, observed during flowering/peak bloom, highlights the salient point that higher leaf transpiration rate provides a protective micro-environment for crucial reproductive organs. This adaptive advantage to cope with unfavorable environmental conditions provides a benefit in terms of lint yield when grown under sufficient irrigation in a hot, arid environment (Figure 2), as more fertilized flowers develop into bolls that are retained through maturation and harvest.

The phenotypic effects of drought and heat stress can further manifest themselves by altering the orientation of leaves that constitute the canopy geometry. Our results show that, for WL plots, NDVI decreases due to leaf wilting over the course of a day (Figure 1), likely in response to low leaf water potential during drought stress (Carmo-Silva *et al.* 2012). The progressive decrease in NDVI tracks directly with the increase in canopy temperature, albeit inversely, over the same time frame. In comparison, NDVI in the WW plots remained high over the course of a day, because canopy geometry was more stable. This was because the leaves of plants under WW conditions were at higher turgor pressure and could maintain a perpendicular orientation to incoming light, but, under WL conditions, the water potential of leaves was reduced, causing a loss of turgor pressure that led to wilting (Zhang *et al.* 2010). Because the angular distribution of leaf area in the canopy was altered to expose more soil in the sensor’s field of view under WL conditions, the amount of near-infrared light reflected was decreased, leading to lower NDVI values. At the whole plant level, LAI (calculated as a function of canopy height and NDVI) followed the same trend of decreasing values for the WL irrigation regime over time, with nonstressed WW plots not changing. Taken together, these results support the hypothesis that the imposed heat and drought stress resulted in in elevated canopy temperature and wilting in a time-dependent manner.

The use of an HTPP system provided us the unique ability to assess variation in canopy traits over plant developmental stages within and across growing seasons. The canopy temperature correlation values presented in Figure 3 (observed for the other HTPP traits as well) demonstrate how the different stages of cotton plant development can be defined by their phenotypic correlations as well as their associated temporal patterns of QTL expression. Although a mapping population of 95 RILs is underpowered for QTL analysis, which limits the ability to detect QTL of small-to-intermediate effect (Xu 2003), its use in this study did not compromise the consistent detection of large effect QTL across plant developmental stages. Canopy temperature exhibited the most dynamic QTL patterns with notable QTL located on chromosomes A05, A09, A12, and D04 that initiated expression at approximately day 216, coinciding with the beginning of boll development and fill (Figure 4). Similar to canopy temperature, QTL associated with NDVI on chromosomes A05, A12, A13, and D04 were detected at approximately day 220. For the remainder of NDVI QTL identified, in addition to those detected for canopy height and LAI, distinct temporal patterns of QTL expression were not as evident. This is most likely due to the inherent relationship between these traits and plant biomass, a property that changes slowly in response to environmental stimuli. Taken together, these findings validate the need for continued phenotyping throughout the season in order to develop a complete picture of abiotic stress response, as no single time point is representative of the diverse physiological processes occurring in the crop. This study also demonstrates the successful implementation of mapping QTL with variable temporal expression. We would have most likely missed capturing these genetic signals, especially for canopy temperature, had traditional single-time point data collection methods been employed.

With respect to applied plant breeding, our results illustrate how HTPP would be a useful tool for evaluating large breeding populations grown under abiotic stress conditions, allowing for the selection of superior genotypes having a favorable array of QTL alleles. We detected significant correlations between HTPP canopy and agronomic traits, with the strength of association dependent on growth stage. For canopy temperature and NDVI, the strongest correlations (*r*^2^) with lint yield were observed during flowering/peak bloom (Figure 2), a growth stage that significantly impacts yield but is also highly susceptible to abiotic stress (Burke and Wanjura 2010; Oosterhuis and Snider 2011; Dabbert and Gore 2014). The QTL on A08, whose allelic effect decreased canopy temperature and maintained NDVI (*i.e.*, less wilting) during this developmental time, and throughout the season, provides an ideal example of QTL that could be selected for to mitigate the effects of unfavorable conditions at critical phenological stages. Although bolls per unit area is the component trait having the strongest relationship with lint yield (Pettigrew 2004), boll size―another trait that has a significant association with lint yield―was also correlated with canopy temperature during flowering/peak bloom. However, boll size also exhibited significant correlations with canopy height and LAI during fiber development and elongation, a key time for fiber quality determination that is also sensitive to high temperatures (Allen and Aleman 2011). For both lint yield and boll size, the observed correlations with HTPP canopy traits were generally the strongest under WL conditions. Given the significant correlations of lint yield and boll size with HTPP canopy traits, the additional use of these HTPP traits as indirect selection criteria in water-limited environments could provide more genetic gain than selection on lint yield and its components alone (Bernardo 2010).

### Conclusion

The phenotyping of plant populations for the study of key agronomic, fiber quality, and physiological traits in cotton has always been a challenge, and with ever increasing population sizes for cotton (Patterson 2012; Abdurakhmonov *et al.* 2014; Tyagi *et al.* 2014), will remain so unless HTPP tools become more prevalent and advanced. The implementation of an HTPP system in conjunction with traditional phenotyping enabled us to study and characterize the response of 95 RILs to the abiotic stresses of heat and drought. Our results demonstrate the ability to map QTL controlling the dynamic response of canopy traits to abiotic stress, and also revealed the temporal nature of QTL expression patterns. Longitudinal phenotypic assessment of canopy traits also provided insight as to which canopy traits are potentially most predictive of lint yield at specific stages of plant development. In summary, our work illustrates how field-based HTPP is a potentially powerful tool to better exploit genomics data for the genetic improvement of crops, which will greatly assist in meeting the challenges facing plant breeding in the 21st century.

## 

## Supplementary Material

Supporting Information
