# Supplementary material for: Field-Based High-Throughput Plant Phenotyping Reveals the Temporal Patterns of Quantitative Trait Loci Associated with Stress-Responsive Traits in Cotton
Source: G3 (Bethesda). 2016 Jan 27;6(4):865–79. doi: 10.1534/g3.115.023515 (PMC4825657; doi:10.1534/g3.115.023515)
Supplement: Supporting Information [file supp_6_4_865__index.html]

Field-Based High-Throughput Plant Phenotyping Reveals the Temporal Patterns of Quantitative Trait Loci Associated with Stress-Responsive Traits in Cotton — Supporting Information 

# Field-Based High-Throughput Plant Phenotyping Reveals the Temporal Patterns of Quantitative Trait Loci Associated with Stress-Responsive Traits in Cotton

## Supporting Information for Pauli *et al.*, 2016

**Files in this Data Supplement:**

- Figure S1 - Plot of daily minimum and maximum air temperatures across the three growing seasons in which the phenotypic data were collected. (.pdf, 351 KB)
- Figure S10 - Box-and-whisker plots of best linear unbiased estimators (BLUEs) for canopy height (m) collected from the TM-1xNM24016 mapping population and its parents across two years under two irrigation regimes, water-limited (WL) and well-watered (WW). (.pdf, 108 KB)
- Figure S11 - Box-and-whisker plots of best linear unbiased estimators (BLUEs) for canopy height (m) collected from the TM-1xNM24016 mapping population and its parents for the 2011 growing season under two irrigation regimes, water-limited (WL) and well-watered (WW). (.pdf, 113 KB)
- Figure S12 - Box-and-whisker plots of best linear unbiased estimators (BLUEs) for canopy height (m) collected from the TM-1xNM24016 mapping population and its parents for the 2012 growing season under two irrigation regimes, water-limited (WL) and well-watered (WW). (.pdf, 114 KB)
- Figure S13 - Box-and-whisker plots of best linear unbiased estimators (BLUEs) for leaf area index (LAI; unitless) collected from the TM-1xNM24016 mapping population and its parents across two years under two irrigation regimes, water-limited (WL) and well-watered (WW). (.pdf, 108 KB)
- Figure S14 - Box-and-whisker plots of best linear unbiased estimators (BLUEs) for leaf area index (LAI; unitless) calculated for the TM-1xNM24016 mapping population and its parents for the 2011 growing season under two irrigation regimes, water-limited (WL) and well-watered (WW). (.pdf, 113 KB)
- Figure S15 - Box-and-whisker plots of best linear unbiased estimators (BLUEs) for leaf area index (LAI; unitless) calculated for the TM-1xNM24016 mapping population and its parents for the 2012 growing season under two irrigation regimes, water-limited (WL) and well-watered (WW). (.pdf, 114 KB)
- Figure S16 - Box-and-whisker plots of estimates of broad-sense heritability (*Ĥ2*) on an entry-mean basis for canopy temperature across three years under two irrigation regimes, water-limited (WL) and well-watered (WW). (.pdf, 141 KB)
- Figure S17 - Box-and-whisker plots of estimates of broad-sense heritability (*Ĥ2*) on an entry-mean basis for normalized difference vegetation index (NDVI) across three years under two irrigation regimes, water-limited (WL) and well-watered (WW). (.pdf, 141 KB)
- Figure S18 - Box-and-whisker plots of estimates of broad-sense heritability (*Ĥ2*) on an entry-mean basis for canopy height across two years under two irrigation regimes, water-limited (WL) and well-watered (WW). (.pdf, 141 KB)
- Figure S19 - Box-and-whisker plots of estimates of broad-sense heritability (*Ĥ2*) on an entry-mean basis for leaf area index (LAI) across two years under two irrigation regimes, water-limited (WL) and well-watered (WW). (.pdf, 141 KB)
- Figure S2 - Box-and-whisker plots of best linear unbiased estimators (BLUEs) for canopy temperature (°C) collected from the TM-1xNM24016 mapping population and its parents across three years under two irrigation regimes, water-limited (WL) and well-watered (WW). (.pdf, 108 KB)
- Figure S20 - Box-and-whisker plots of best linear unbiased estimators (BLUEs) for plant height (m) collected from the TM-1xNM24016 mapping population and its parents across three years under two irrigation regimes, water-limited (WL) and well-watered (WW). (.pdf, 108 KB)
- Figure S21 - Box-and-whisker plots of best linear unbiased estimators (BLUEs) for plant height (m) collected from the TM-1xNM24016 mapping population and its parents for the 2010 growing season under two irrigation regimes, water-limited (WL) and well-watered (WW). (.pdf, 111 KB)
- Figure S22 - Box-and-whisker plots of best linear unbiased estimators (BLUEs) for plant height (m) collected from the TM-1xNM24016 mapping population and its parents for the 2011 growing season under two irrigation regimes, water-limited (WL) and well-watered (WW). (.pdf, 112 KB)
- Figure S23 - Box-and-whisker plots of best linear unbiased estimators (BLUEs) for plant height (m) collected from the TM-1xNM24016 mapping population and its parents for the 2012 growing season under two irrigation regimes, water-limited (WL) and well-watered (WW). (.pdf, 113 KB)
- Figure S24 - Box-and-whisker plots of estimates of broad-sense heritability (*Ĥ2*) on an entry-mean basis for plant height across three years under two irrigation regimes, water-limited (WL) and well-watered (WW). (.pdf, 141 KB)
- Figure S25 - Genome-wide scan for quantitative trait loci (QTL) associated with normalized difference vegetation index (NDVI) across three years under two irrigation regimes, water-limited (WL, top panel) and well-watered (WW, bottom panel). (.pdf, 678 KB)
- Figure S26 - Genome-wide scan for quantitative trait loci (QTL) associated with canopy height across two years under two irrigation regimes, water-limited (WL, top panel) and well-watered (WW, bottom panel). (.pdf, 711 KB)
- Figure S27 - Genome-wide scan for quantitative trait loci (QTL) associated with leaf area index (LAI) across two years under two irrigation regimes, water-limited (WL, top panel) and well-watered (WW, bottom panel). (.pdf, 752 KB)
- Table S1 - Fixed effects for environmental parameters. (.pdf, 103 KB)
- Table S2 - Summary of meteorological conditions. (.pdf, 113 KB)
- Figure S3 - Box-and-whisker plots of best linear unbiased estimators (BLUEs) for canopy temperature (°C) collected from the TM-1xNM24016 mapping population and its parents for the 2010 growing season under two irrigation regimes, water-limited (WL) and well-watered (WW). (.pdf, 111 KB)
- Table S3 - Fixed effects for canopy temperature. (.pdf, 113 KB)
- Table S4 - Summary information for canopy temperature in 2010. (.pdf, 113 KB)
- Table S5 - Summary information for canopy temperature in 2011. (.pdf, 134 KB)
- Table S6 - Summary information for canopy temperature in 2012. (.pdf, 134 KB)
- Table S7 - Fixed effects for NDVI. (.pdf, 116 KB)
- Table S8 - Summary information for NDVI in 2010. (.pdf, 113 KB)
- Table S9 - Summary information for NDVI in 2011. (.pdf, 126 KB)
- Table S10 - Summary information for NDVI in 2012. (.pdf, 129 KB)
- Table S11 - Summary information for canopy height in 2011. (.pdf, 122 KB)
- Table S12 - Summary information for canopy height in 2012. (.pdf, 126 KB)
- Figure S4 - Box-and-whisker plots of best linear unbiased estimators (BLUEs) for canopy temperature (°C) collected from the TM-1xNM24016 mapping population and its parents for the 2011 growing season under two irrigation regimes, water-limited (WL) and well-watered (WW). (.pdf, 113 KB)
- Table S13 - Fixed effects for canopy height. (.pdf, 113 KB)
- Table S14 - Fixed effects for LAI. (.pdf, 112 KB)
- Table S15 - Summary information for LAI in 2011. (.pdf, 122 KB)
- Table S16 - Summary information for LAI in 2012. (.pdf, 130 KB)
- Table S17 - Heritability estimates for canopy temperature. (.pdf, 168 KB)
- Table S18 - Heritability estimates for NDVI. (.pdf, 169 KB)
- Table S19 - Heritability estimates for canopy height. (.pdf, 166 KB)
- Table S20 - Heritability estimates for LAI. (.pdf, 166 KB)
- Table S21 - Fixed effects for agronomic, fiber quality, and physiological traits. (.pdf, 118 KB)
- Table S22 - Fixed effects for plant height. (.pdf, 110 KB)
- Figure S5 - Box-and-whisker plots of best linear unbiased estimators (BLUEs) for canopy temperature (°C) collected from the TM-1xNM24016 mapping population and its parents for the 2012 growing season under two irrigation regimes, water-limited (WL) and well-watered (WW). (.pdf, 114 KB)
- Table S23 - Summary information for plant height in 2010. (.pdf, 105 KB)
- Table S24 - Summary information for plant height in 2011. (.pdf, 116 KB)
- Table S25 - Summary information for plant height in 2012. (.pdf, 116 KB)
- Table S26 - Heritability estimates for plant height. (.pdf, 171 KB)
- Table S27 - Fixed effects for physiological traits within years. (.pdf, 115 KB)
- Table S28 - Summary of the non-linear relationship between canopy temperature and lint yield across cotton plant growth stages. (.pdf, 179 KB)
- Table S29 - Summary of QTL for canopy temperature. (.pdf, 124 KB)
- Table S30 - Summary of QTL for NDVI. (.pdf, 125 KB)
- Table S31 - Summary of QTL for canopy height. (.pdf, 124 KB)
- Table S32 - Summary of QTL for plant height. (.pdf, 116 KB)
- Figure S6 - Box-and-whisker plots of best linear unbiased estimators (BLUEs) for normalized difference vegetation index (NDVI; unitless) collected from the TM-1xNM24016 mapping population and its parents across three years under two irrigation regimes, water-limited (WL) and well-watered (WW). (.pdf, 108 KB)
- Table S33 - Summary of QTL for LAI. (.pdf, 126 KB)
- Table S34 - Summary of QTL for agronomic, fiber quality and physiological traits. (.pdf, 138 KB)
- Figure S7 - Box-and-whisker plots of best linear unbiased estimators (BLUEs) for normalized difference vegetation index (NDVI; unitless) collected from the TM-1xNM24016 mapping population and its parents for the 2010 growing season under two irrigation regimes, water-limited (WL) and well-watered (WW). (.pdf, 111 KB)
- Figure S8 - Box-and-whisker plots of best linear unbiased estimators (BLUEs) for normalized difference vegetation index (NDVI; unitless) collected from the TM-1xNM24016 mapping population and its parents for the 2011 growing season under two irrigation regimes, water-limited (WL) and well-watered (WW). (.pdf, 113 KB)
- Figure S9 - Box-and-whisker plots of best linear unbiased estimators (BLUEs) for normalized difference vegetation index (NDVI; unitless) collected from the TM-1xNM24016 mapping population and its parents for the 2012 growing season under two irrigation regimes, water-limited (WL) and well-watered (WW). (.pdf, 113 KB)
- File S1 - Best linear unbiased estimators (BLUEs) for non-high throughput plant phenotyping (HTPP) and HTPP traits. (.xlsx, 1498 KB)
- File S2 - Marker genotype data from the TM-1xNM24016 mapping population. (.xlsx, 299 KB)
- File S3 - Integration of the TM-1xNM24016 genetic linkage map with the *G. hirsutum* L. acc. TM-1 draft genome sequence. (.xlsx, 79 KB)
- File S4 - Trait correlations. The Microsoft Excel file contains Pearson correlation coefficients with corresponding raw *P*-values reported below their respective correlation coefficient. (.xlsx, 529 KB)
