# Supplementary material for: Field-Based High-Throughput Plant Phenotyping Reveals the Temporal Patterns of Quantitative Trait Loci Associated with Stress-Responsive Traits in Cotton
Source: G3 (Bethesda). 2016 Jan 27;6(4):865–79. doi: 10.1534/g3.115.023515 (PMC4825657; doi:10.1534/g3.115.023515)
Supplement: Supporting Information [file supp_g3.115.023515_FigureS1.pdf]

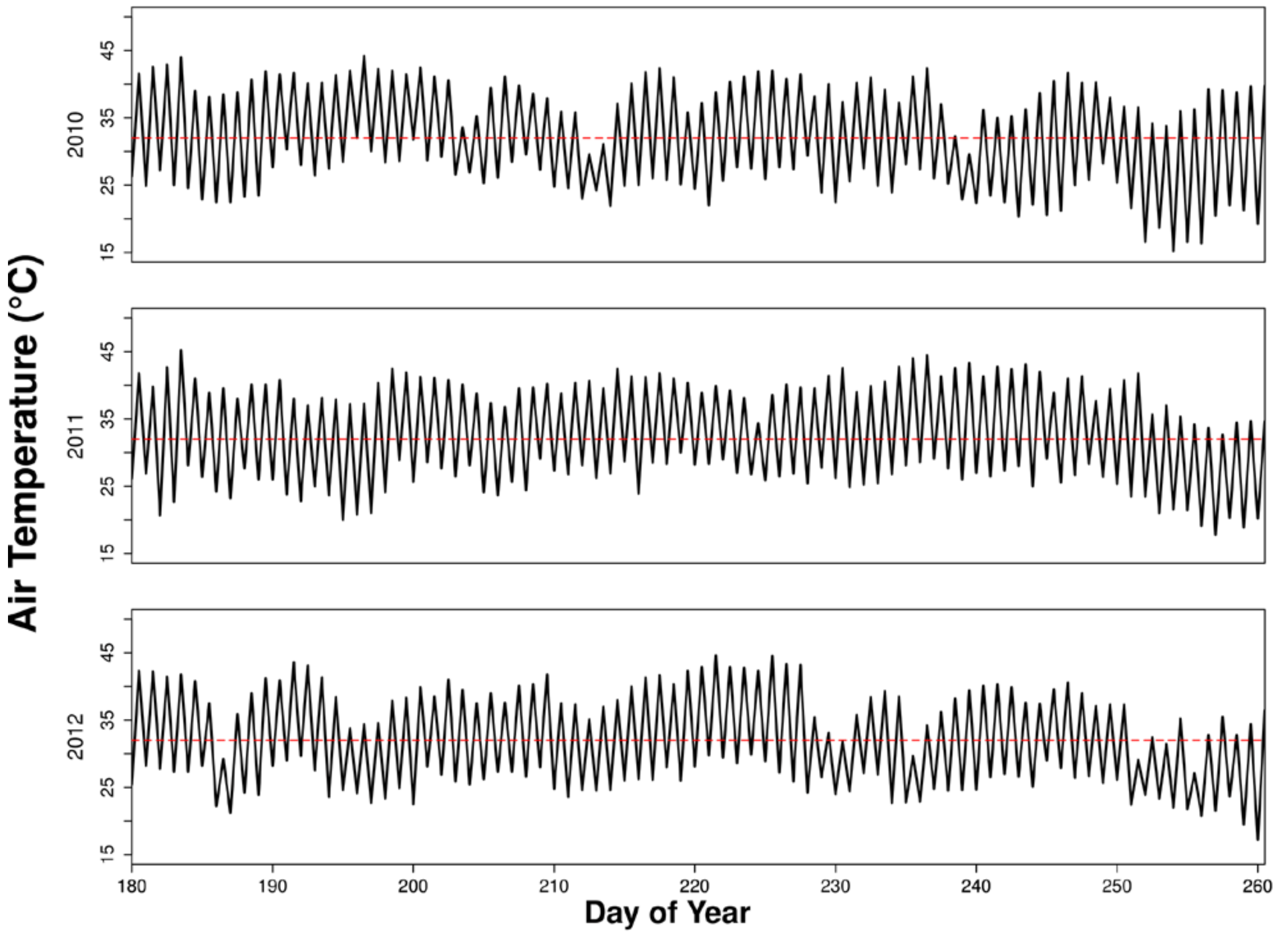

Figure S1 Plot of daily minimum and maximum air temperatures across the three growing seasons in which the phenotypic data were collected. The x-axis denotes the day of year (Julian calendar) in which phenotypic data were collected, with data collection typically beginning shortly after day 180 and concluding at approximately day 260. The red, dashed line, at 32°C, represents the temperature threshold above which lint yields are sharply decreased per Schlenker and Roberts (2009).
