# Supplementary material for: Field-Based High-Throughput Plant Phenotyping Reveals the Temporal Patterns of Quantitative Trait Loci Associated with Stress-Responsive Traits in Cotton
Source: G3 (Bethesda). 2016 Jan 27;6(4):865–79. doi: 10.1534/g3.115.023515 (PMC4825657; doi:10.1534/g3.115.023515)
Supplement: Supporting Information [file supp_g3.115.023515_FigureS13.pdf]

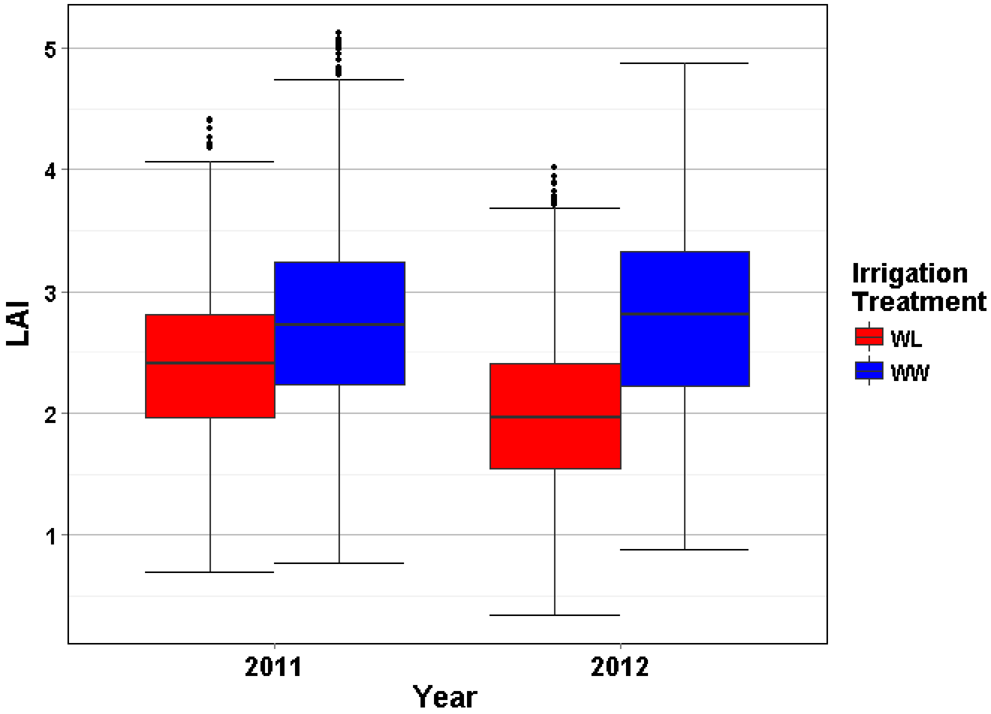


**Figure S13** Box-and-whisker plots of best linear unbiased estimators (BLUEs) for leaf area index (LAI; unitless) collected from the TM-1×NM24016 mapping population and its parents across two years under two irrigation regimes, water-limited (WL) and well-watered (WW). The total number of times that the high-throughput plant phenotyping (HTPP) system was driven over the entire set of experimental plots to collect canopy height and NDVI data to calculate LAI was 18 and 25 for 2011 and 2012, respectively. The horizontal black line inside the box is the median.
