# Supplementary material for: Field-Based High-Throughput Plant Phenotyping Reveals the Temporal Patterns of Quantitative Trait Loci Associated with Stress-Responsive Traits in Cotton
Source: G3 (Bethesda). 2016 Jan 27;6(4):865–79. doi: 10.1534/g3.115.023515 (PMC4825657; doi:10.1534/g3.115.023515)
Supplement: Supporting Information [file supp_g3.115.023515_FigureS17.pdf]

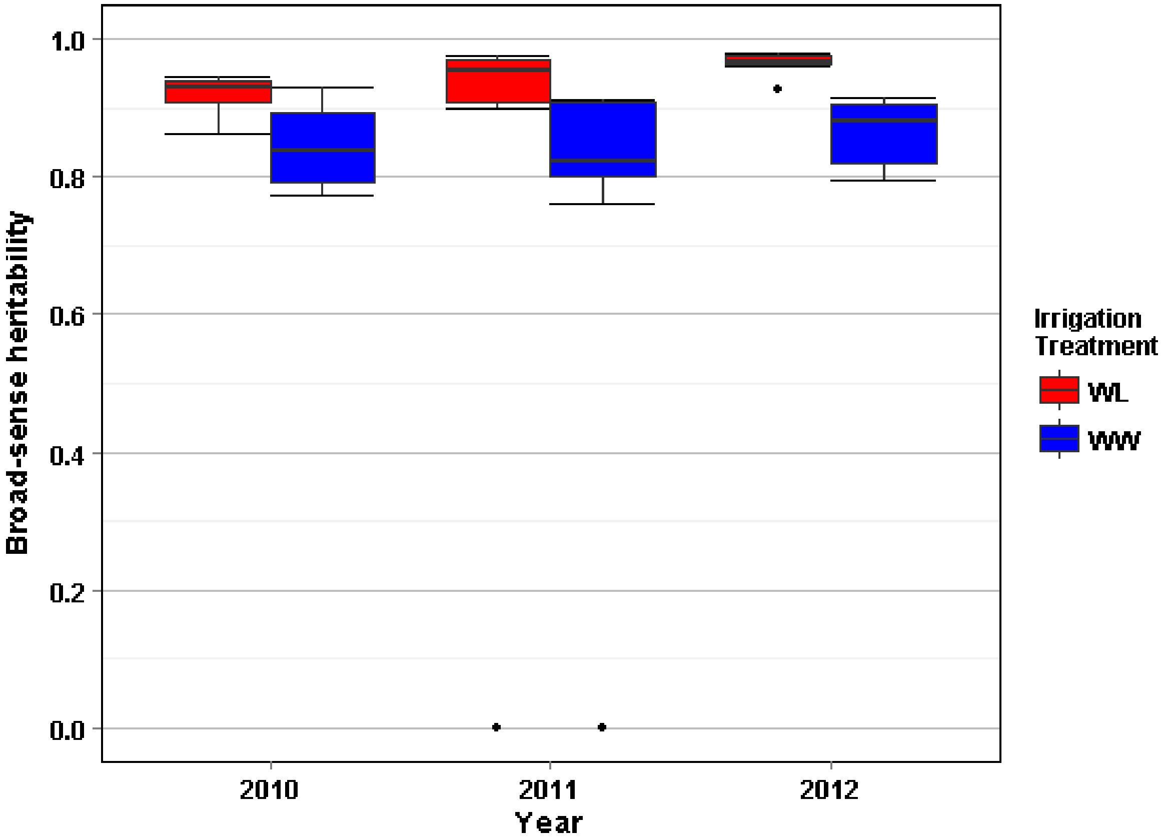

Figure S17 Box-and-whisker plots of estimates of broad-sense heritability ($\hat{H}^2$) on an entry-mean basis for normalized difference vegetation index (NDVI) across three years under two irrigation regimes, water-limited (WL) and well-watered (WW). The total number of times that the high-throughput plant phenotyping (HTPP) system was driven over the entire set of experimental plots to collect NDVI data was 10, 22, and 25, for 2010, 2011, and 2012, respectively. The horizontal black line inside the box is the median.
