# Supplementary material for: Field-Based High-Throughput Plant Phenotyping Reveals the Temporal Patterns of Quantitative Trait Loci Associated with Stress-Responsive Traits in Cotton
Source: G3 (Bethesda). 2016 Jan 27;6(4):865–79. doi: 10.1534/g3.115.023515 (PMC4825657; doi:10.1534/g3.115.023515)
Supplement: Supporting Information [file supp_g3.115.023515_FigureS20.pdf]

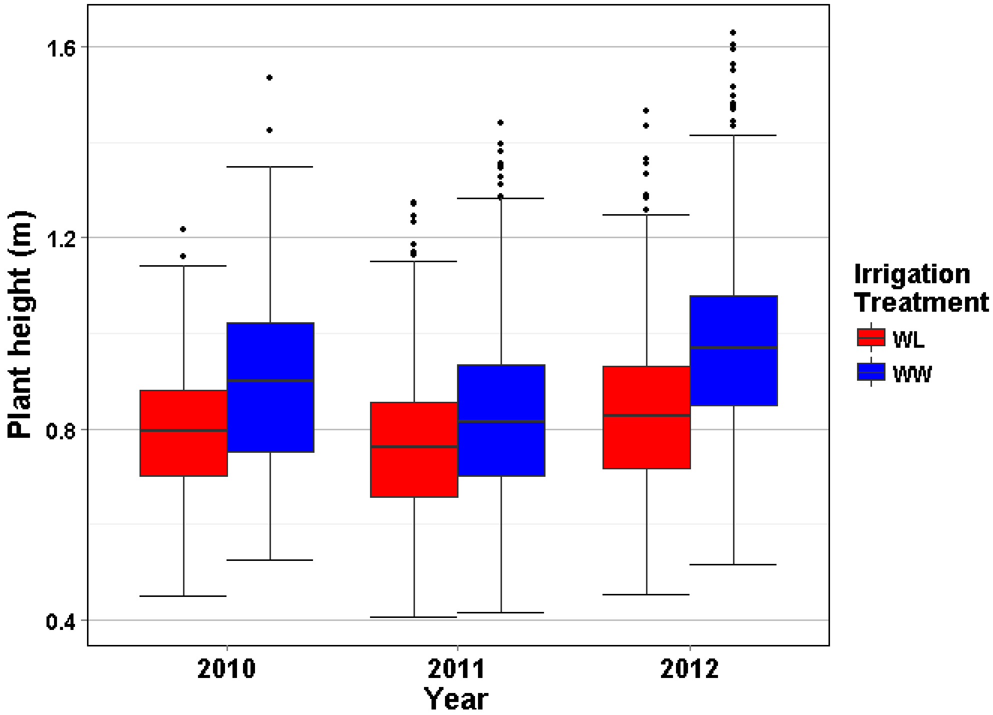

Figure S20 Box-and-whisker plots of best linear unbiased estimators (BLUEs) for plant height (m) collected from the TM-1×NM24016 mapping population and its parents across three years under two irrigation regimes, water-limited (WL) and well-watered (WW). The total number of different days that plant height data was manually collected from the entire set of experimental plots was 3, 11, and 11 for 2010, 2011, and 2012, respectively. The horizontal black line inside the box is the median.
