# Supplementary material for: Field-Based High-Throughput Plant Phenotyping Reveals the Temporal Patterns of Quantitative Trait Loci Associated with Stress-Responsive Traits in Cotton
Source: G3 (Bethesda). 2016 Jan 27;6(4):865–79. doi: 10.1534/g3.115.023515 (PMC4825657; doi:10.1534/g3.115.023515)
Supplement: Supporting Information [file supp_g3.115.023515_FigureS24.pdf]

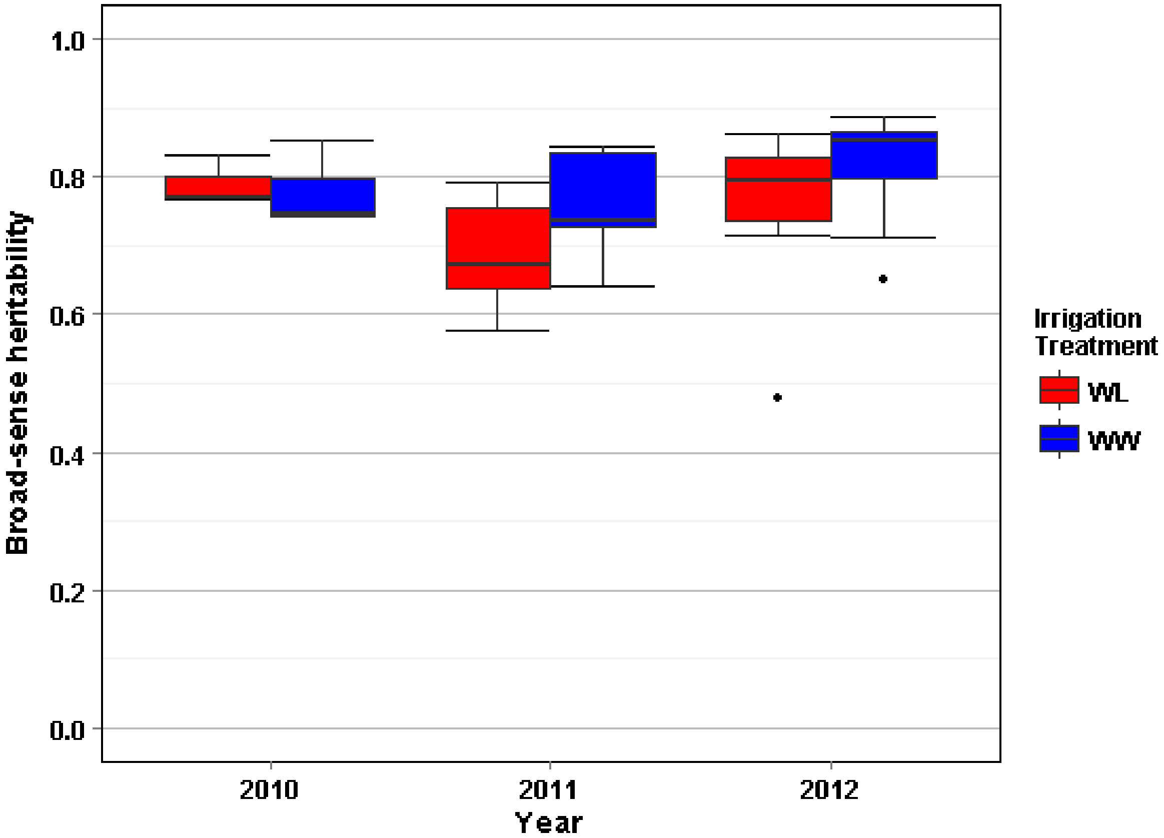

Figure S24 Box-and-whisker plots of estimates of broad-sense heritability ($\hat{H}^2$) on an entry-mean basis for plant height across three years under two irrigation regimes, water-limited (WL) and well-watered (WW). The total number of different days that plant height data was manually collected from the entire set of experimental plots was 3, 11, and 11 for 2010, 2011, and 2012, respectively.
