# Supplementary material for: Field-Based High-Throughput Plant Phenotyping Reveals the Temporal Patterns of Quantitative Trait Loci Associated with Stress-Responsive Traits in Cotton
Source: G3 (Bethesda). 2016 Jan 27;6(4):865–79. doi: 10.1534/g3.115.023515 (PMC4825657; doi:10.1534/g3.115.023515)
Supplement: Supporting Information [file supp_g3.115.023515_FigureS25.pdf]

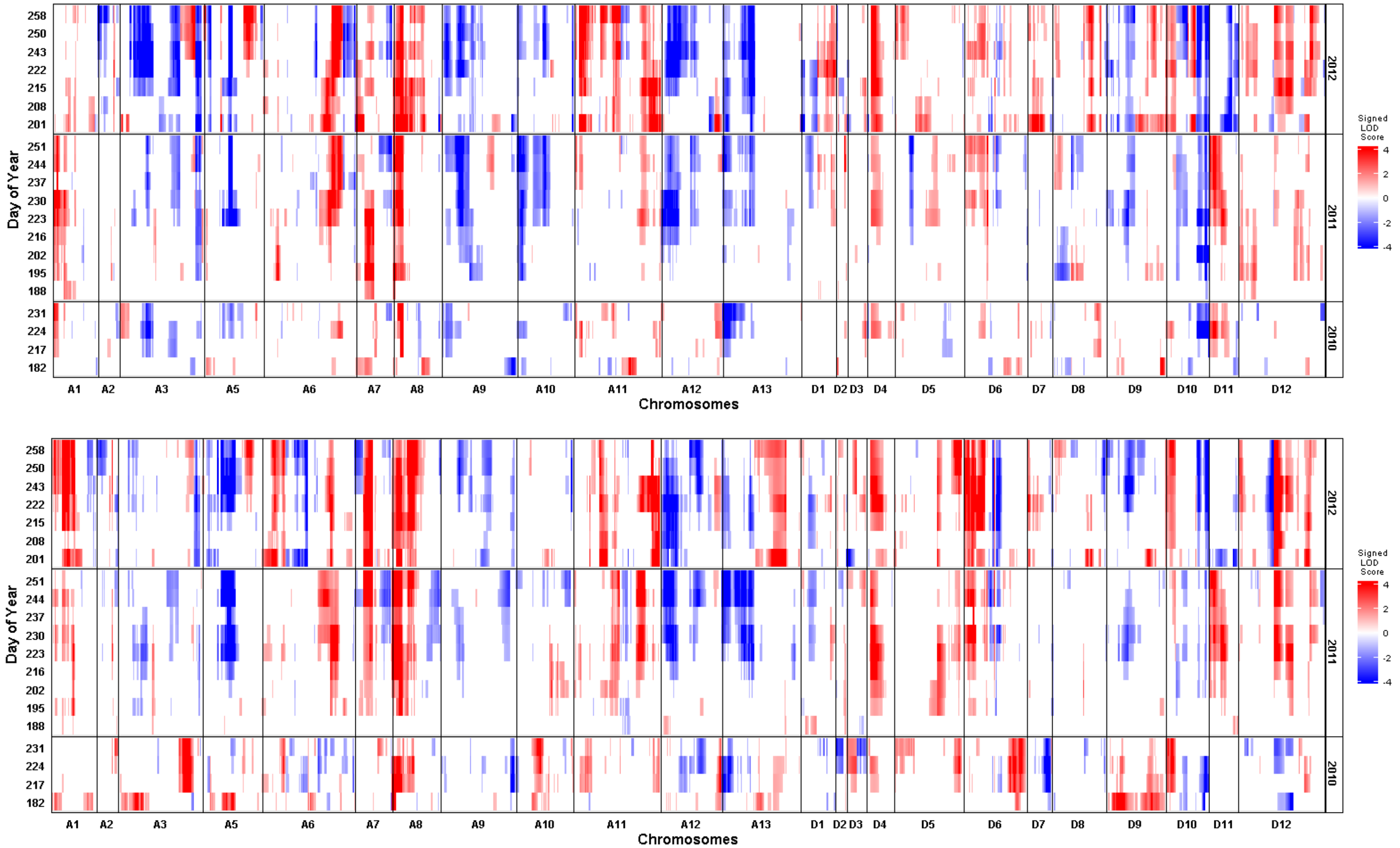

Figure S25 Genome-wide scan for quantitative trait loci (QTL) associated with normalized difference vegetation index (NDVI) across three years under two irrigation regimes, water-limited (WL, top panel) and well-watered (WW, bottom panel). Color is representative of the estimated additive allelic effect, with red and blue colors indicating increased and decreased NDVI, respectively, when substituting a NM24016 allele with an allele from TM-1. The intensity of the color represents the magnitude of the logarithm of odds (LOD) score with 3.3 as the significance threshold to declare QTL – values above 3.3 were assigned a value of 4 for presentation purposes. The left-hand y-axis denotes day of the growing season (Julian calendar) that data were collected, and the right-hand y-axis denotes year. The x-axis represents the mapped chromosomes of tetraploid cotton with marker intervals in centimorgans (cM). Chromosomes A04 and D13 did not have any mapped markers.
