# Supplementary material for: Field-Based High-Throughput Plant Phenotyping Reveals the Temporal Patterns of Quantitative Trait Loci Associated with Stress-Responsive Traits in Cotton
Source: G3 (Bethesda). 2016 Jan 27;6(4):865–79. doi: 10.1534/g3.115.023515 (PMC4825657; doi:10.1534/g3.115.023515)
Supplement: Supporting Information [file supp_g3.115.023515_TableS1.pdf]

Table S1 Fixed effects for environmental parameters. F values for fixed effects from an analysis of variance (ANOVA) for key environmental parameters over the time frame in which phenotypic data were collected in a growing season (Phenotyping Season), spanning days 180 through 260 (Julian calendar), and for the specific days on which data were collected using the high-throughput plant phenotyping (HTPP) system. The model used for analyzing the phenotyping season included the grand mean and year as fixed effects. The model used for analyzing specific days on which HTPP data were collected included the grand mean, year, and day nested within year as fixed effects. The environmental parameter data used for this analysis were collected hourly from 0700-1600 MST from 2010-12, as this encompasses the time when the HTPP platform would be used in the field. Precipitation on all days in which HTPP data were collected was 0 mm. Meteorological data were collected by the AZMET weather station located at the Maricopa Agricultural Center of the University of Arizona located in Maricopa, AZ.

**Phenotyping Season**	**Parameter**	**Days HTPP Data Collected**	
**Year**		Year	Day*Year
**13.70********	**Air temperature**	4.32*	2.22**
**3.93***	**Evapotranspiration**	0.58 ^{NS}	0.55 ^{NS}
**5.09****	**Precipitation**	NA	NA
**124.10********	**Relative humidity**	2.85*	5.34***
**0.89** ^{NS}	**Solar radiation**	0.59 ^{NS}	0.35 ^{NS}
**47.18********	**Vapor pressure deficit**	5.03**	2.40**

NS Not Significant at the < 0.05 level.
* Significant at the < 0.05 level.
** Significant at the < 0.01 level.
*** Significant at the < 0.001 level.
**** Significant at the < 0.0001 level.
