# Supplementary material for: Field-Based High-Throughput Plant Phenotyping Reveals the Temporal Patterns of Quantitative Trait Loci Associated with Stress-Responsive Traits in Cotton
Source: G3 (Bethesda). 2016 Jan 27;6(4):865–79. doi: 10.1534/g3.115.023515 (PMC4825657; doi:10.1534/g3.115.023515)
Supplement: Supporting Information [file supp_g3.115.023515_TableS2.pdf]

Table S2 Summary of meteorological conditions. Summary of weather conditions from days on which data were collected using the HTPP system. The summarized environmental parameter data were collected hourly from 0700-1600 MST from 2010-12, as this encompasses the time when the HTPP system would be used in the field. The meteorological data were collected by the AZMET weather station located at the Maricopa Agricultural Center of the University of Arizona located in Maricopa, AZ.

Year	DOY[a]	Air temp. (C°)	RH (%)[b]	VPD (kPa)[c]	Solar rad. (MJ/m^2)[d]	Precip. (mm)	10.2 cm soil temp (C°)	Ave. wind speed (m/s)	Ref ET (mm)[e]
**2010**	182	38.39	21.19	5.50	2.44	0	30.65	4.51	0.77
	217	36.61	22.58	5.14	2.44	0	34.25	1.30	0.64
	224	36.52	22.58	4.96	2.36	0	35.88	2.18	0.64
	231	35.29	39.75	3.78	2.29	0	30.97	0.95	0.56
**2011**	188	35.17	33.88	3.92	2.46	0	33.39	2.49	0.65
	195	31.58	14.54	4.19	2.67	0	29.90	2.78	0.74
	202	35.35	28.29	4.33	2.20	0	33.35	2.18	0.58
	216	34.95	35.86	3.92	2.36	0	33.93	1.51	0.60
	223	33.01	42.26	3.00	2.27	0	32.76	1.62	0.56
	230	37.72	27.05	5.05	2.21	0	33.37	2.33	0.62
	237	36.98	25.65	4.89	1.94	0	35.29	2.17	0.55
	244	35.63	29.70	4.53	2.14	0	32.66	1.51	0.57
	251	35.17	23.91	4.72	2.19	0	32.04	1.27	0.57
**2012**	201	35.71	19.67	4.83	1.88	0	32.35	2.89	0.55
	208	35.31	24.96	4.52	2.33	0	34.30	2.87	0.66
	215	34.34	31.00	3.99	2.42	0	32.33	1.82	0.63
	222	37.76	27.70	4.94	2.36	0	35.01	1.85	0.64
	243	33.13	43.80	3.02	2.12	0	32.85	2.34	0.55
	250	33.21	47.08	2.85	1.91	0	34.18	1.49	0.47
	258	29.20	30.15	2.92	2.12	0	27.50	5.85	0.66

a. DOY, day of year - Julian calendar.
b. RH, relative humidity.
c. VPD, vapor pressure deficit.
d. Solar rad, solar radiation.
e. Ref ET, reference evapotranspiration using Penman-Monteith method.
