# Supplementary material for: Field-Based High-Throughput Plant Phenotyping Reveals the Temporal Patterns of Quantitative Trait Loci Associated with Stress-Responsive Traits in Cotton
Source: G3 (Bethesda). 2016 Jan 27;6(4):865–79. doi: 10.1534/g3.115.023515 (PMC4825657; doi:10.1534/g3.115.023515)
Supplement: Supporting Information [file supp_g3.115.023515_TableS3.pdf]

Table S3 Fixed effects for canopy temperature. F values for fixed effects from an analysis of variance (ANOVA) for the TM-1×NM24106 recombinant inbred line (RIL) population, its two parents, and commercial check varieties for canopy temperature collected from 2010-12 at the Maricopa Agricultural Center located in Maricopa, AZ.

Year	DOY[a]	Source						
		TOD[b]	Genotype	Irrigation Regime	Genotype* Irrigation Regime	TOD*Genotype	TOD* Irrigation Regime	TOD*Genotype* Irrigation Regime
**2010**	217	82.84 ***	5.28 ****	15.46 *	1.55 **	4.76 ****	11.26 *	1.73 **
	224	89.31 ****	3.13 ****	27.99 **	1.40 *	3.82 ****	6.05 *	1.67 ****
	231	470.40 ****	3.43 ****	5.75 *	1.17 NS	2.24 ****	3.61 *	1.15 NS
**2011**	188	1009.00 ****	4.37 ****	0.08 NS	0.68 NS	3.01 ****	1.53 NS	0.7 NS
	195	4.34 ****	73.76 ****	0.80 NS	8.21 ****	0.28 NS	0.84 NS	0.00 NS
	202	208.00 ****	3.04 ****	53.97 ****	0.82 NS	3.44 ****	14.55 ***	1.38 NS
	216	357.40 ****	4.58 ****	29.04 ****	0.81 NS	4.51 ****	0.20 NS	0.90 NS
	223	574.50 ****	8.01 ****	49.21 ****	1.09 NS	5.74 ****	2.08 NS	0.99 NS
	230	147.00 ****	7.79 ****	36.68 ***	1.32 NS	6.46 ****	3.32 NS	1.44 **
	237	59.88 ***	4.98 ****	24.30 **	1.41 *	4.56 ****	2.65 NS	1.79 ****
	244	104.60 ****	4.52 ****	54.46 ***	1.45 *	3.73 ****	4.28 NS	1.36 **
	251	231.60 ****	4.68 ****	53.43 ***	1.44 *	4.65 ****	7.62 *	1.51 ***
**2012**	201	64.75 ****	2.25 ****	105.00 ****	1.38 *	2.68 ****	4.95 *	1.57 ****
	208	12.26 *	4.03 ****	12.68 *	1.73 ***	2.09 ****	1.03 NS	0.98 NS
	215	7.01 NS	4.51 ****	6.65 NS	1.58 **	2.20 ****	0.50 NS	0.99 NS
	222	218.60 ****	2.84 ****	210.40 ****	1.16 NS	4.07 ****	26.31 ***	1.92 ****
	243	145.30 ****	4.29 ****	56.47 ****	1.21 NS	4.25 ****	8.96 **	1.60 ****
	250	205.10 ****	2.28 ****	150.40 ****	1.20 NS	3.22 ****	22.57 ***	1.65 ****
	258	296.60 ****	2.95 ****	7.29 *	0.94 NS	3.42 ****	3.67 NS	1.18 NS

a. DOY, day of year – Julian calendar.
b. TOD, time of day within the day of year – MST.
NS Not Significant at the < 0.05 level.
* Significant at the < 0.05 level.
** Significant at the < 0.01 level.
*** Significant at the < 0.001 level.
**** Significant at the < 0.0001 level.
