# Supplementary material for: Field-Based High-Throughput Plant Phenotyping Reveals the Temporal Patterns of Quantitative Trait Loci Associated with Stress-Responsive Traits in Cotton
Source: G3 (Bethesda). 2016 Jan 27;6(4):865–79. doi: 10.1534/g3.115.023515 (PMC4825657; doi:10.1534/g3.115.023515)
Supplement: Supporting Information [file supp_g3.115.023515_TableS4.pdf]

Table S4 Summary information for canopy temperature in 2010. Canopy temperature means, standard deviations, midparent values, and ranges of best linear unbiased estimators (BLUEs) for the TM-1×NM24106 recombinant inbred line (RIL) population and its two parents under two irrigation regimes, water-limited (WL) and well-watered (WW), in Maricopa, AZ in 2010.

**DOY**[a]	**TOD**[b]	**Irrigation Regime**	**Parents**			**RIL population**			
			**TM-1**	**NM24016**	**Midparent**	**Mean**	**Std. Dev**	**Min.**	**Max.**
**217**	0700	WL	27.84	28.31	28.07	28.51	0.59	27.31	30.24
		WW	27.93	27.88	27.90	28.07	0.41	27.17	29.43
	1300	WL	36.24	38.27	37.26	37.84	1.73	34.62	45.02
		WW	31.77	32.94	32.35	32.35	0.81	30.13	35.09
**224**	0700	WL	26.17	26.81	26.49	26.84	0.44	26.20	28.32
		WW	25.29	26.10	25.70	26.08	0.39	25.22	27.30
	1000	WL	32.49	33.31	32.90	33.25	1.11	31.26	37.44
		WW	29.86	30.37	30.12	30.37	0.66	28.95	32.49
	1300	WL	38.56	39.67	39.11	38.59	1.73	35.05	45.14
		WW	32.89	33.15	33.02	32.93	0.95	30.97	35.59
**231**	0700	WL	27.21	27.59	27.40	27.39	0.22	26.78	28.08
		WW	27.38	27.57	27.47	27.58	0.22	27.08	28.24
	1000	WL	33.45	33.08	33.26	33.01	0.75	31.49	35.57
		WW	33.10	32.57	32.83	32.52	0.73	30.89	33.90
	1300	WL	33.79	33.65	33.72	33.40	0.85	31.86	36.12
		WW	32.47	32.56	32.51	32.47	0.72	30.71	34.64

a. DOY, day of year – Julian calendar.
b. TOD, time of day within the day of year – MST..
