# Supplementary material for: Field-Based High-Throughput Plant Phenotyping Reveals the Temporal Patterns of Quantitative Trait Loci Associated with Stress-Responsive Traits in Cotton
Source: G3 (Bethesda). 2016 Jan 27;6(4):865–79. doi: 10.1534/g3.115.023515 (PMC4825657; doi:10.1534/g3.115.023515)
Supplement: Supporting Information [file supp_g3.115.023515_TableS5.pdf]

Table S5 Summary information for canopy temperature in 2011. Canopy temperature means, standard deviations, midparent values, and ranges of best linear unbiased estimators (BLUEs) for the TM-1×NM24106 recombinant inbred line (RIL) population and its two parents under two irrigation regimes, water-limited (WL) and well-watered (WW), in Maricopa, AZ in 2011.

DOY[a]	TOD[b]	Irrigation Regime	Parents			RIL population			
			TM-1	NM24016	Midparent	Mean	Std. Dev	Min.	Max.
188	0700	WL	30.76	32.50	31.63	31.98	0.88	30.28	34.23
		WW	30.23	30.99	30.61	31.44	0.72	29.86	33.68
	1300	WL	40.82	43.73	42.28	42.57	1.87	38.22	47.23
		WW	41.05	42.60	41.82	43.00	1.68	38.40	46.63
195	0700	WL	27.15	29.18	28.17	28.13	0.83	26.39	30.69
		WW	24.65	26.03	25.34	25.80	0.69	24.28	28.04
	1500	WL	34.80	36.77	35.78	35.93	0.96	33.97	38.45
		WW	31.36	34.01	32.68	33.25	0.97	31.15	35.61
202	0700	WL	32.17	33.63	32.90	32.96	1.11	30.21	35.55
		WW	30.18	32.60	31.39	31.00	0.93	29.11	33.46
	1300	WL	41.56	43.56	42.56	42.39	1.96	37.33	46.97
		WW	34.92	36.76	35.84	36.75	1.80	32.99	40.46
216	0700	WL	28.74	29.82	29.28	29.30	0.51	28.21	30.73
		WW	27.91	28.13	28.02	28.22	0.41	27.36	29.40
	1100	WL	34.89	36.08	35.49	35.27	1.30	32.37	38.60
		WW	33.02	34.69	33.86	33.85	1.24	31.50	37.54
	1500	WL	35.11	36.79	35.95	35.92	1.33	32.44	39.64
		WW	33.74	35.36	34.55	34.60	1.37	31.36	39.46
223	0700	WL	27.80	28.50	28.15	28.10	0.49	27.12	29.45
		WW	27.15	27.17	27.16	27.29	0.38	26.36	28.25
	1100	WL	34.78	35.21	35.00	34.62	1.43	31.84	38.84
		WW	33.04	33.18	33.11	33.02	1.19	30.61	36.04
	1500	WL	33.81	35.30	34.55	34.71	1.47	31.21	39.00
		WW	33.41	33.94	33.67	33.33	1.17	30.85	36.51
230	0700	WL	29.24	28.99	29.11	29.23	0.43	28.32	30.37
		WW	28.09	28.04	28.07	28.09	0.34	27.41	29.12
	1100	WL	38.68	38.50	38.59	38.58	1.69	34.96	42.83
		WW	36.20	36.04	36.12	35.70	1.51	32.97	39.30
	1500	WL	37.43	37.60	37.51	37.76	1.11	35.00	41.00
		WW	33.60	34.38	33.99	33.82	1.30	31.55	38.38
237	0700	WL	28.44	28.37	28.41	28.50	0.40	27.49	29.33
		WW	27.03	27.22	27.13	26.89	0.39	26.07	28.21
	1100	WL	41.17	41.02	41.09	40.75	1.37	37.42	44.23
		WW	34.97	34.51	34.74	34.73	1.40	32.44	39.21
	1500	WL	39.18	39.72	39.45	39.40	0.95	37.37	41.86
		WW	34.67	34.65	34.66	34.76	1.13	32.55	38.89
244	0700	WL	27.64	27.82	27.73	27.88	0.41	27.06	28.95
		WW	25.73	25.95	25.84	25.87	0.41	25.08	27.08
	1100	WL	38.98	38.91	38.95	38.98	1.21	36.04	41.87
		WW	33.30	32.86	33.08	33.41	1.30	30.95	37.64
	1500	WL	39.48	40.00	39.74	39.63	0.97	37.26	41.49
		WW	33.37	33.29	33.33	33.50	1.26	30.86	38.06
251	0700	WL	24.98	25.33	25.15	25.32	0.36	24.49	26.45
		WW	24.03	24.16	24.09	24.13	0.33	23.49	24.95
	1100	WL	34.33	34.63	34.48	34.54	1.01	32.22	37.33
		WW	31.71	31.85	31.78	32.08	1.08	30.13	36.25
	1500	WL	35.27	36.31	35.79	35.84	0.72	34.22	37.78
		WW	30.38	31.26	30.82	31.13	1.01	29.40	35.01

a. DOY, day of year – Julian calendar.
b. TOD, time of day within the day of year – MST.
