# Supplementary material for: Field-Based High-Throughput Plant Phenotyping Reveals the Temporal Patterns of Quantitative Trait Loci Associated with Stress-Responsive Traits in Cotton
Source: G3 (Bethesda). 2016 Jan 27;6(4):865–79. doi: 10.1534/g3.115.023515 (PMC4825657; doi:10.1534/g3.115.023515)
Supplement: Supporting Information [file supp_g3.115.023515_TableS6.pdf]

Table S6 Summary information for canopy temperature in 2012. Canopy temperature means, standard deviations, midparent values, and ranges of best linear unbiased estimators (BLUEs) for the TM-1×NM24106 recombinant inbred line (RIL) population and its two parents under two irrigation regimes, water-limited (WL) and well-watered (WW), in Maricopa, AZ in 2012.

**DOY**[a]	**TOD**[b]	**Irrigation Regime**	**Parents**			**RIL population**			
			TM-1	NM24016	Midparent	Mean	Std. Dev	Min.	Max.
**201**	0700	WL	27.77	28.63	28.20	28.36	0.65	26.43	29.93
		WW	25.64	26.37	26.00	26.22	0.69	24.92	27.87
	1000	WL	40.17	39.96	40.06	40.51	1.98	36.02	46.02
		WW	31.46	34.16	32.81	33.35	1.78	29.95	41.46
	1300	WL	37.52	37.72	37.62	38.00	1.44	35.02	41.77
		WW	30.06	31.85	30.96	31.11	1.12	29.02	35.67
	1500	WL	36.59	37.02	36.80	36.66	1.00	34.88	39.66
		WW	30.24	31.49	30.86	31.15	0.93	29.52	34.78
**208**	1000	WL	32.25	32.74	32.49	33.07	1.45	30.38	37.63
		WW	29.36	31.42	30.39	30.44	1.14	28.75	34.40
	1500	WL	37.07	37.09	37.08	37.37	1.70	34.45	43.84
		WW	31.40	33.60	32.50	32.89	1.28	29.63	37.39
**215**	1000	WL	33.01	33.13	33.07	33.13	1.19	30.24	36.39
		WW	31.23	31.93	31.58	31.32	1.03	29.21	35.42
	1300	WL	35.97	35.57	35.77	36.23	1.77	32.63	40.84
		WW	32.33	33.71	33.02	33.09	1.55	30.41	39.75
**222**	0700	WL	28.60	28.47	28.53	28.98	0.55	27.78	30.45
		WW	28.06	28.05	28.06	27.97	0.56	26.72	29.05
	1000	WL	38.15	37.01	37.58	37.43	1.45	34.31	42.66
		WW	32.75	33.24	32.99	32.96	1.23	31.12	37.69
	1300	WL	47.75	45.08	46.42	45.26	2.45	39.78	52.64
		WW	35.70	36.70	36.20	35.76	1.99	32.33	43.14
	1500	WL	46.37	44.26	45.32	44.36	1.93	40.20	49.88
		WW	35.34	36.15	35.75	35.87	1.52	31.96	41.80
**243**	0700	WL	26.86	26.62	26.74	27.02	0.37	26.13	27.88
		WW	27.11	26.81	26.96	26.88	0.34	26.29	27.63
	1000	WL	31.90	31.34	31.62	31.61	0.94	29.49	33.97
		WW	30.15	30.04	30.09	30.02	0.77	28.79	32.43
	1300	WL	36.93	36.18	36.55	36.19	1.67	33.05	40.20
		WW	33.28	33.49	33.39	32.93	1.41	30.60	37.21
	1500	WL	36.04	35.68	35.86	35.42	1.27	33.10	38.36
		WW	31.68	31.55	31.62	31.53	1.08	29.30	34.79
**250**	0700	WL	26.75	26.72	26.73	27.06	0.42	26.18	27.96
		WW	27.14	26.72	26.93	26.75	0.41	25.88	27.69
	1000	WL	34.41	34.15	34.28	34.25	1.04	32.14	36.98
		WW	31.80	30.93	31.36	31.16	0.95	29.30	34.91
	1300	WL	41.47	41.45	41.46	40.73	1.66	37.49	44.57
		WW	34.42	34.08	34.25	33.74	1.57	31.06	40.04
	1500	WL	34.64	35.20	34.92	34.81	0.96	32.86	36.96
		WW	29.81	29.58	29.70	29.81	0.78	28.41	32.30
**258**	0700	WL	19.89	20.00	19.95	20.54	0.31	19.73	21.39
		WW	21.10	20.95	21.03	20.99	0.28	20.30	21.71
	1000	WL	26.63	26.53	26.58	27.07	0.49	26.10	28.26
		WW	26.86	26.57	26.72	26.71	0.44	25.88	27.89
	1300	WL	30.41	30.41	30.41	30.66	0.85	29.24	32.71
		WW	29.27	29.10	29.18	29.22	0.84	27.83	31.76
	1500	WL	29.32	28.99	29.15	29.42	0.70	27.87	31.09
		WW	27.99	27.94	27.97	28.01	0.66	26.50	29.74

a. DOY, day of year – Julian calendar.

b. TOD, time of day within the day of year – MST.
