# Supplementary material for: Field-Based High-Throughput Plant Phenotyping Reveals the Temporal Patterns of Quantitative Trait Loci Associated with Stress-Responsive Traits in Cotton
Source: G3 (Bethesda). 2016 Jan 27;6(4):865–79. doi: 10.1534/g3.115.023515 (PMC4825657; doi:10.1534/g3.115.023515)
Supplement: Supporting Information [file supp_g3.115.023515_TableS7.pdf]

Table S7 Fixed effects for NDVI. F values for fixed effects from an analysis of variance (ANOVA) for the TM-1×NM24106 recombinant inbred line (RIL) population, its two parents, and commercial check varieties collected from 2010-12 at the Maricopa agricultural Center located in Maricopa, AZ.

**Year**	**DOY**[a]	**Source**						
		TOD[b]	Genotype	Irrigation Regime	Genotype* Irrigation Regime	TOD*Genotype	TOD* Irrigation Regime	TOD*Genotype* Irrigation Regime
**2010**	182	0.07 NS	3.68 ****	1.91 NS	0.85 NS	1.83 ***	0.00 NS	0.62 NS
	217	21.59 ****	3.14 ****	308.50 ****	1.73 ***	3.57 ****	66.16 ****	2.42 ****
	224	10.23 ****	3.02 ****	240.60 ****	1.96 ****	3.71 ****	24.14 ****	2.38 ****
	231	2.20 NS	2.09 ****	92.58 ****	1.56 **	1.32 *	5.12 **	1.04 NS
**2011**	188	0.61 NS	2.21 ****	0.12 NS	0.93 NS	3.70 ****	0.00 NS	1.20 NS
	195	0.91 NS	3.00 ****	0.75 NS	0.84 NS	4.71 ****	0.16 NS	1.27 NS
	202	32.12 ****	2.39 ****	64.97 ****	0.93 NS	5.52 ****	47.69 ****	1.46 NS
	216	539.20 ****	2.79 ****	24.95 ****	0.83 NS	2.73 ****	11.44 ***	0.77 NS
	223	284.10 ****	4.24 ****	47.14 ****	0.80 NS	2.61 ****	20.56 ****	1.26 *
	230	0.07 NS	8.43 ****	2.43 NS	1.12 NS	3.21 ****	0.08 NS	1.27 *
	237	0.65 NS	6.91 ****	5.39 NS	1.40 *	2.65 ****	0.62 NS	1.54 **
	244	0.05 NS	6.33 ****	13.48 **	1.31 *	2.77 ****	0.47 NS	2.05 ****
	251	0.79 NS	6.84 ****	15.68 **	1.30 NS	2.78 ****	0.09 NS	1.28 *
**2012**	201	0.82 NS	2.32 ****	13.27 **	1.20 NS	3.08 ****	0.42 NS	1.61 ****
	208	0.00 NS	4.18 ****	7.59 *	1.70 ***	2.53 ****	0.03 NS	1.17 NS
	215	0.24 NS	2.77 ****	13.15 *	1.05 NS	2.04 ****	0.01 NS	1.18 NS
	222	1.97 NS	3.06 ****	73.26 ****	1.16 NS	4.87 ****	2.05 NS	3.28 ****
	243	0.10 NS	4.39 ****	19.49 **	1.16 NS	1.99 ****	0.03 NS	1.13 NS
	250	1.08 NS	3.52 ****	37.03 ***	1.10 NS	3.12 ****	1.05 NS	2.24 ****
	258	0.01 NS	4.08 ****	7.86 *	1.11 NS	2.69 ****	0.02 NS	1.32 **

a. DOY, day of year – Julian calendar.
b. TOD, time of day within the day of year – MST.
NS Not Significant at the < 0.05 level.
* Significant at the < 0.05 level.
** Significant at the < 0.01 level.
*** Significant at the < 0.001 level.
**** Significant at the < 0.0001 level.
