# Supplementary material for: Field-Based High-Throughput Plant Phenotyping Reveals the Temporal Patterns of Quantitative Trait Loci Associated with Stress-Responsive Traits in Cotton
Source: G3 (Bethesda). 2016 Jan 27;6(4):865–79. doi: 10.1534/g3.115.023515 (PMC4825657; doi:10.1534/g3.115.023515)
Supplement: Supporting Information [file supp_g3.115.023515_TableS8.pdf]

Table S8 Summary information for NDVI in 2010. Normalized difference vegetation index (NDVI) means, standard deviations, midparent values, and ranges of best linear unbiased estimators (BLUEs) for the TM-1×NM24106 recombinant inbred line (RIL) population and its two parents under two irrigation regimes, water-limited (WL) and well-watered (WW), in Maricopa, AZ in 2010.

DOY[a]	TOD[b]	Irrigation Regime	Parents			RIL population			
			**TM-1**	**NM24016**	**Midparent**	**Mean**	**Std. Dev**	**Min.**	**Max.**
**182**	0700	WL	0.20	0.22	0.21	0.19	0.03	0.12	0.26
		WW	0.15	0.17	0.16	0.17	0.02	0.12	0.24
	0900	WL	0.20	0.21	0.21	0.18	0.03	0.12	0.25
		WW	0.15	0.17	0.16	0.17	0.03	0.08	0.23
**217**	0700	WL	0.72	0.72	0.72	0.70	0.06	0.39	0.81
		WW	0.75	0.77	0.76	0.78	0.03	0.69	0.84
	1300	WL	0.67	0.71	0.69	0.67	0.08	0.31	0.79
		WW	0.76	0.79	0.77	0.78	0.03	0.68	0.85
**224**	0700	WL	0.75	0.74	0.75	0.73	0.05	0.52	0.81
		WW	0.76	0.78	0.77	0.79	0.02	0.71	0.84
	1000	WL	0.74	0.74	0.74	0.73	0.05	0.51	0.80
		WW	0.77	0.79	0.78	0.79	0.02	0.73	0.84
	1300	WL	0.69	0.72	0.70	0.70	0.07	0.38	0.79
		WW	0.77	0.80	0.78	0.79	0.03	0.73	0.85
**231**	0700	WL	0.78	0.78	0.78	0.78	0.03	0.65	0.82
		WW	0.78	0.80	0.79	0.80	0.02	0.74	0.85
	1000	WL	0.78	0.79	0.79	0.78	0.04	0.63	0.84
		WW	0.79	0.81	0.80	0.81	0.02	0.74	0.86
	1300	WL	0.78	0.79	0.78	0.77	0.04	0.62	0.83
		WW	0.79	0.81	0.80	0.81	0.02	0.75	0.86

a. DOY, day of year – Julian calendar.
b. TOD, time of day within the day of year – MST.
