# Supplementary material for: Field-Based High-Throughput Plant Phenotyping Reveals the Temporal Patterns of Quantitative Trait Loci Associated with Stress-Responsive Traits in Cotton
Source: G3 (Bethesda). 2016 Jan 27;6(4):865–79. doi: 10.1534/g3.115.023515 (PMC4825657; doi:10.1534/g3.115.023515)
Supplement: Supporting Information [file supp_g3.115.023515_TableS10.pdf]

Table S10 Summary information for NDVI in 2012. Normalized difference vegetation index (NDVI) means, standard deviations, midparent values, and ranges of best linear unbiased estimators (BLUEs) for the TM-1×NM24106 recombinant inbred line (RIL) population and its two parents under two irrigation regimes, water-limited (WL) and well-watered (WW), in Maricopa, AZ in 2012.

DOY[a]	TOD[b]	Irrigation Regime	Parents			RIL population			
			TM-1	NM24016	Midparent	Mean	Std. Dev	Min.	Max.
201	0700	WL	0.62	0.61	0.62	0.58	0.06	0.41	0.71
		WW	0.70	0.62	0.66	0.66	0.07	0.44	0.81
	1000	WL	0.52	0.53	0.52	0.48	0.07	0.28	0.64
		WW	0.70	0.62	0.66	0.65	0.08	0.43	0.82
	1300	WL	0.60	0.60	0.60	0.55	0.07	0.35	0.69
		WW	0.72	0.64	0.68	0.67	0.07	0.45	0.82
	1500	WL	0.62	0.62	0.62	0.58	0.06	0.38	0.72
		WW	0.72	0.64	0.68	0.68	0.07	0.45	0.82
208	1000	WL	0.65	0.66	0.66	0.64	0.06	0.49	0.77
		WW	0.78	0.73	0.76	0.75	0.05	0.55	0.84
	1300	WL	0.65	0.66	0.66	0.63	0.07	0.44	0.77
		WW	0.79	0.75	0.77	0.75	0.05	0.56	0.84
215	0700	WL	0.70	0.73	0.72	0.69	0.06	0.53	0.80
		WW	0.81	0.79	0.80	0.80	0.04	0.63	0.85
	1000	WL	0.72	0.74	0.73	0.71	0.05	0.55	0.82
		WW	0.82	0.81	0.81	0.81	0.04	0.63	0.86
	1300	WL	0.73	0.76	0.74	0.72	0.05	0.57	0.82
		WW	0.83	0.81	0.82	0.82	0.04	0.66	0.87
222	0700	WL	0.68	0.74	0.71	0.69	0.06	0.52	0.82
		WW	0.79	0.79	0.79	0.79	0.05	0.61	0.85
	1000	WL	0.61	0.72	0.66	0.66	0.07	0.49	0.81
		WW	0.80	0.80	0.80	0.80	0.05	0.60	0.86
	1300	WL	0.48	0.64	0.56	0.60	0.08	0.40	0.76
		WW	0.79	0.80	0.79	0.79	0.05	0.60	0.86
	1500	WL	0.46	0.64	0.55	0.59	0.09	0.37	0.77
		WW	0.79	0.80	0.80	0.79	0.05	0.60	0.86
243	0700	WL	0.71	0.78	0.75	0.74	0.05	0.60	0.84
		WW	0.78	0.79	0.79	0.80	0.04	0.66	0.85
	1000	WL	0.71	0.78	0.75	0.73	0.05	0.59	0.84
		WW	0.78	0.80	0.79	0.80	0.04	0.65	0.86
	1300	WL	0.70	0.79	0.75	0.74	0.05	0.59	0.85
		WW	0.79	0.80	0.80	0.81	0.04	0.66	0.86
	1500	WL	0.70	0.79	0.74	0.74	0.05	0.60	0.84
		WW	0.79	0.81	0.80	0.81	0.04	0.66	0.86
250	0700	WL	0.70	0.77	0.73	0.71	0.05	0.59	0.81
		WW	0.75	0.76	0.75	0.77	0.05	0.60	0.84
	1000	WL	0.69	0.75	0.72	0.70	0.05	0.55	0.81
		WW	0.75	0.77	0.76	0.77	0.05	0.62	0.84
	1300	WL	0.62	0.68	0.65	0.65	0.07	0.46	0.78
		WW	0.74	0.76	0.75	0.77	0.06	0.54	0.83
	1500	WL	0.66	0.73	0.70	0.69	0.06	0.52	0.80
		WW	0.76	0.77	0.76	0.78	0.05	0.59	0.84
258	0700	WL	0.76	0.80	0.78	0.76	0.04	0.67	0.84
		WW	0.76	0.78	0.77	0.79	0.04	0.63	0.85
	1000	WL	0.76	0.79	0.78	0.76	0.04	0.66	0.83
		WW	0.77	0.78	0.77	0.79	0.05	0.61	0.85
	1300	WL	0.75	0.80	0.78	0.75	0.04	0.65	0.83
		WW	0.77	0.78	0.78	0.79	0.05	0.60	0.85
	1500	WL	0.75	0.80	0.78	0.75	0.04	0.65	0.84
		WW	0.77	0.78	0.77	0.79	0.05	0.61	0.85

a. DOY, day of year – Julian calendar.
b. TOD, time of day within the day of year – MST.
