# Supplementary material for: Field-Based High-Throughput Plant Phenotyping Reveals the Temporal Patterns of Quantitative Trait Loci Associated with Stress-Responsive Traits in Cotton
Source: G3 (Bethesda). 2016 Jan 27;6(4):865–79. doi: 10.1534/g3.115.023515 (PMC4825657; doi:10.1534/g3.115.023515)
Supplement: Supporting Information [file supp_g3.115.023515_TableS13.pdf]

Table S13 Fixed effects for canopy height. F values for fixed effects from an analysis of variance (ANOVA) for the TM-1×NM24106 recombinant inbred line (RIL) population, its two parents, and commercial check varieties collected in 2011 and 2012 at the Maricopa Agricultural Center located Maricopa, AZ.

Year	DOY[a]	Source						
		TOD[b]	Genotype	Irrigation Regime	Genotype* Irrigation Regime	TOD*Genotype	TOD* Irrigation Regime	TOD*Genotype* Irrigation Regime
**2011**	202	6.59 *	3.71 ****	46.55 ****	1.06 NS	1.25 NS	3.89 NS	0.82 NS
	216	0.09 NS	5.82 ****	6.50 NS	1.18 NS	1.36 NS	0.02 NS	0.93 NS
	223	3.85 *	11.72 ****	33.24 ****	1.34 *	1.02 NS	3.96 *	0.99 NS
	230	0.26 NS	13.90 ****	2.62 NS	1.43 **	1.54 ***	0.88 NS	1.18 *
	237	0.01 NS	16.20 ****	8.31 *	1.58 **	1.15 NS	0.28 NS	0.80 NS
	244	0.36 NS	9.56 ****	12.90 **	1.21 NS	1.31 ***	0.62 NS	1.29 **
	251	0.47 NS	10.56 ****	77.47 ****	1.11 NS	1.08 NS	1.43 NS	1.15 NS
**2012**	201	0.34 NS	4.37 ****	5.20 NS	1.28 NS	1.01 NS	0.10 NS	1.04 NS
	208	0.01 NS	6.26 ****	11.73 *	1.07 NS	1.34 *	0.02 NS	1.33 *
	215	0.09 NS	9.45 ****	203.00 ****	1.25 NS	1.18 NS	0.30 NS	1.17 NS
	222	4.55 **	9.76 ****	467.00 ****	1.21 NS	1.36 ***	0.36 NS	1.05 NS
	243	3.91 *	16.06 ****	212.40 ****	1.63 **	1.11 NS	0.76 NS	1.14 NS
	250	4.26 **	11.79 ****	339.30 ****	1.50 **	1.16 NS	0.82 NS	1.08 NS
	258	6.89 ***	12.06 ****	43.36 ****	1.40 *	1.14 NS	0.09 NS	1.27 **

a. DOY, day of year – Julian calendar.
b. TOD, time of day within the day of year – MST.
NS Not Significant at the < 0.05 level.
* Significant at the < 0.05 level.
** Significant at the < 0.01 level.
*** Significant at the < 0.001 level.
**** Significant at the < 0.0001 level.
