# Supplementary material for: Field-Based High-Throughput Plant Phenotyping Reveals the Temporal Patterns of Quantitative Trait Loci Associated with Stress-Responsive Traits in Cotton
Source: G3 (Bethesda). 2016 Jan 27;6(4):865–79. doi: 10.1534/g3.115.023515 (PMC4825657; doi:10.1534/g3.115.023515)
Supplement: Supporting Information [file supp_g3.115.023515_TableS14.pdf]

Table S14 Fixed effects for LAI. F values for fixed effects from an analysis of variance (ANOVA) for the TM-1×NM24106 recombinant inbred line (RIL) population, its two parents, and commercial check varieties for leaf area index (LAI) calculated for 2011 and 2012 using data collected at the Maricopa Agricultural Center located in Maricopa, AZ.

Year	DOY[a]	Source						
		TOD[b]	Genotype	Irrigation Regime	Genotype* Irrigation Regime	TOD*Genotype	TOD* Irrigation Regime	TOD*Genotype* Irrigation Regime
2011	202	1.29 NS	2.60 ****	87.35 ****	0.93 NS	2.07 ****	47.39 ****	0.99 NS
	216	0.52 NS	3.91 ****	8.06 *	0.99 NS	1.46 *	0.02 NS	0.87 NS
	223	0.51 NS	7.64 ****	17.30 **	1.11 NS	1.02 NS	0.99 NS	0.91 NS
	230	0.01 NS	11.97 ****	5.34 NS	1.33 *	1.71 ****	0.37 NS	1.22 *
	237	0.10 NS	13.83 ****	7.48 *	1.57 **	1.13 NS	0.45 NS	0.84 NS
	244	0.22 NS	8.47 ****	14.56 **	1.27 NS	1.44 **	0.36 NS	1.29 *
	251	1.54 NS	8.56 ****	128.70 ****	1.10 NS	1.08 NS	0.36 NS	1.11 NS
2012	201	0.33 NS	2.95 ****	14.51 **	1.32 *	1.35 ***	0.13 NS	1.21 *
	208	0.00 NS	3.51 ****	11.21 *	1.12 NS	1.53 **	0.03 NS	1.46 **
	215	0.07 NS	5.17 ****	48.06 ***	1.12 NS	1.05 NS	0.01 NS	1.05 NS
	222	1.13 NS	6.75 ****	149.30 ****	1.24 NS	1.60 ****	0.68 NS	1.15 NS
	243	1.76 NS	13.85 ****	286.40 ****	1.50 **	1.20 *	0.78 NS	1.18 *
	250	18.41 ****	9.18 ****	427.10 ****	1.41 *	1.12 NS	9.73 ****	1.03 NS
	258	5.48 **	11.75 ****	57.59 ****	1.39 *	1.15 NS	0.08 NS	1.30 **

a. DOY, day of year – Julian calendar.
b. TOD, time of day within the day of year – MST.
NS Not Significant at the < 0.05 level.
* Significant at the < 0.05 level.
** Significant at the < 0.01 level.
*** Significant at the < 0.001 level.
**** Significant at the < 0.0001 level.
