# Supplementary material for: Field-Based High-Throughput Plant Phenotyping Reveals the Temporal Patterns of Quantitative Trait Loci Associated with Stress-Responsive Traits in Cotton
Source: G3 (Bethesda). 2016 Jan 27;6(4):865–79. doi: 10.1534/g3.115.023515 (PMC4825657; doi:10.1534/g3.115.023515)
Supplement: Supporting Information [file supp_g3.115.023515_TableS15.pdf]

Table S15 Summary information for LAI in 2011. Leaf area index (LAI) means, standard deviations, midparent values, and ranges of best linear unbiased estimators (BLUEs) for the TM-1×NM24106 recombinant inbred line (RIL) population and its two parents under two irrigation regimes, water-limited (WL) and well-watered (WW), in Maricopa, AZ in 2011.

DOY[a]	TOD[b]	Irrigation Regime	Parents			RIL population			
			TM-1	NM24016	Midparent	Mean	Std. Dev	Min.	Max.
202	0700	WL	1.65	1.26	1.45	1.59	0.34	0.90	2.68
		WW	2.09	1.86	1.97	1.83	0.40	0.77	2.80
	1300	WL	1.46	1.09	1.28	1.43	0.35	0.69	2.37
		WW	2.10	2.07	2.08	1.93	0.41	1.00	2.82
216	1100	WL	2.03	1.82	1.93	2.12	0.43	1.23	3.42
		WW	2.51	2.45	2.48	2.43	0.52	1.36	3.73
	1500	WL	2.18	1.90	2.04	2.19	0.43	1.25	3.42
		WW	2.58	2.55	2.57	2.53	0.53	1.27	3.86
223	0700	WL	1.99	2.00	2.00	2.27	0.47	1.36	3.67
		WW	2.45	2.68	2.57	2.64	0.58	1.50	4.41
	1100	WL	2.19	2.17	2.18	2.35	0.45	1.46	3.71
		WW	2.40	2.67	2.53	2.71	0.58	1.38	4.36
	1500	WL	2.21	2.27	2.24	2.36	0.48	1.33	3.79
		WW	2.21	2.47	2.34	2.55	0.59	1.08	4.16
230	0700	WL	2.25	2.63	2.44	2.66	0.55	1.58	4.41
		WW	2.28	2.68	2.48	2.78	0.66	1.40	4.71
	1100	WL	2.24	2.62	2.43	2.54	0.54	1.28	4.19
		WW	2.56	2.70	2.63	2.87	0.69	1.47	4.67
	1500	WL	2.23	2.62	2.43	2.54	0.55	1.27	4.21
		WW	2.55	2.72	2.63	2.86	0.70	1.42	4.64
237	1100	WL	2.22	2.51	2.37	2.41	0.55	1.16	4.27
		WW	2.48	2.72	2.60	2.89	0.67	1.45	4.82
	1500	WL	2.25	2.58	2.42	2.56	0.52	1.38	4.07
		WW	2.40	2.85	2.62	2.85	0.65	1.46	4.73
244	0700	WL	2.46	2.63	2.54	2.62	0.52	1.53	3.79
		WW	2.64	2.87	2.76	3.02	0.71	1.64	4.95
	1100	WL	2.38	2.65	2.51	2.57	0.55	1.41	4.20
		WW	2.65	2.72	2.68	2.95	0.70	1.52	4.83
	1500	WL	2.23	2.60	2.42	2.56	0.57	1.30	4.40
		WW	2.77	2.99	2.88	3.16	0.71	1.72	5.12
251	0700	WL	2.57	2.82	2.69	2.73	0.52	1.62	4.19
		WW	2.90	3.18	3.04	3.21	0.70	1.70	5.07
	1100	WL	2.75	2.74	2.75	2.78	0.50	1.67	4.18
		WW	3.20	2.95	3.08	3.24	0.67	1.67	5.04
	1500	WL	2.49	2.73	2.61	2.79	0.53	1.65	4.34
		WW	3.10	2.92	3.01	3.22	0.69	1.56	5.05

a. DOY, day of year – Julian calendar.
b. TOD, time of day within the day of year – MST.
