# Supplementary material for: Field-Based High-Throughput Plant Phenotyping Reveals the Temporal Patterns of Quantitative Trait Loci Associated with Stress-Responsive Traits in Cotton
Source: G3 (Bethesda). 2016 Jan 27;6(4):865–79. doi: 10.1534/g3.115.023515 (PMC4825657; doi:10.1534/g3.115.023515)
Supplement: Supporting Information [file supp_g3.115.023515_TableS19.pdf]

Table S19 Heritability estimates for canopy height. Estimates of broad-sense heritability ($\hat{H}^2$) on an entry-mean basis for canopy height for the TM-1×NM24106 recombinant inbred line (RIL) population evaluated under two irrigation regimes, water-limited (WL) and well-watered (WW) conditions.

Year	DOY[a]	Irrigation Regime	$\hat{H}^2$
2011	202	WL	0.85
		WW	0.78
	216	WL	0.92
		WW	0.89
	223	WL	0.95
		WW	0.93
	230	WL	0.94
		WW	0.94
	237	WL	0.96
		WW	0.95
	244	WL	0.95
		WW	0.93
	251	WL	0.95
		WW	0.93
2012	201	WL	0.85
		WW	0.78
	208	WL	0.88
		WW	0.84
	215	WL	0.92
		WW	0.89
	222	WL	0.96
		WW	0.95
	243	WL	0.96
		WW	0.95
	250	WL	0.95
		WW	0.95
	258	WL	0.94
		WW	0.95

a. DOY, Day of year – Julian calendar.
