# Supplementary material for: Field-Based High-Throughput Plant Phenotyping Reveals the Temporal Patterns of Quantitative Trait Loci Associated with Stress-Responsive Traits in Cotton
Source: G3 (Bethesda). 2016 Jan 27;6(4):865–79. doi: 10.1534/g3.115.023515 (PMC4825657; doi:10.1534/g3.115.023515)
Supplement: Supporting Information [file supp_g3.115.023515_TableS21.pdf]

Table S21 Fixed effects for agronomic, fiber quality, and physiological traits. F values for fixed effects from an analysis of variance (ANOVA) for the TM-1×NM24106 recombinant inbred line (RIL) population, its two parents, and commercial check varieties for physiological, fiber quality, and agronomic traits. Data were collected for years 2010-12 at the Maricopa Agricultural Center located in Maricopa, AZ.

Trait	Source		
	Genotype	Irrigation Regime	Genotype*Irrigation Regime
**ABA conc.**	1.73****	4.97 ^{NS}	1.05 ^{NS}
**$\Delta^{13}C$**	3.68****	2.37 ^{NS}	1.62***
**Sugar conc.**	1.41*	3.41 ^{NS}	0.89 ^{NS}
**Fiber elongation**	44.56****	0.77 ^{NS}	1.19 ^{NS}
**Fiber micronaire**	34.36****	0.08 ^{NS}	1.32*
**Fiber length (upper half mean)**	27.16****	25.35****	1.12 ^{NS}
**Fiber uniformity**	17.75****	1.06 ^{NS}	0.98 ^{NS}
**Fiber Strength**	26.79****	0.97 ^{NS}	1.03 ^{NS}
**Boll size**	21.03****	63.87****	1.22 ^{NS}
**Lint yield**	17.48****	51.22****	1.61***
**Seed per boll**	14.39****	28.51****	1.27*

Figure S16
nt at the < 0.05
level.
* Significant at the < 0.05 level.
** Significant at the < 0.01 level.
*** Significant at the < 0.001 level.
**** Significant at the < 0.0001 level.
