# Supplementary material for: Field-Based High-Throughput Plant Phenotyping Reveals the Temporal Patterns of Quantitative Trait Loci Associated with Stress-Responsive Traits in Cotton
Source: G3 (Bethesda). 2016 Jan 27;6(4):865–79. doi: 10.1534/g3.115.023515 (PMC4825657; doi:10.1534/g3.115.023515)
Supplement: Supporting Information [file supp_g3.115.023515_TableS22.pdf]

Table S22 Fixed effects for plant height. F values for fixed effects from an analysis of variance (ANOVA) for the TM-1×NM24106 recombinant inbred line (RIL) population, its two parents, and commercial check varieties for plant height collected form 2010-12 at the Maricopa Agricultural Center located in Maricopa, AZ.

Year	DOY[a]	Source		
		Genotype	Irrigation Regime	Genotype* Irrigation Regime
**2010**	208	5.64 ****	7.10 *	1.42 *
	218	6.47 ****	8.09 NS	0.93 NS
	342	10.23 ****	129.40 ****	1.36 *
**2011**	189	4.28 ****	2.01 NS	1.00 NS
	195	4.93 ****	0.63 NS	1.23 NS
	203	4.08 ****	6.12 *	1.44 *
	209	4.94 ****	31.33 ****	0.95 NS
	216	4.79 ****	13.87 ***	1.06 NS
	223	6.87 ****	4.06 *	1.13 NS
	231	7.37 ****	0.79 NS	1.30 NS
	238	7.04 ****	187.00 ****	1.30 NS
	244	7.09 ****	0.23 NS	1.94 ****
	251	6.29 ****	7.87 NS	1.12 NS
	299	6.11 ****	3.71 NS	1.55 **
**2012**	201	3.52 ****	0.05 NS	0.85 NS
	209	5.43 ****	62.86 ****	0.99 NS
	215	6.65 ****	151.30 ****	1.09 NS
	223	8.31 ****	146.70 ****	1.12 NS
	233	11.75 ****	138.70 ****	1.20 NS
	240	10.43 ****	97.04 ****	1.33 *
	243	11.69 ****	59.02 ****	1.18 NS
	250	9.93 ****	86.73 ****	1.69 ***
	257	9.01 ****	4.70 NS	1.48 *
	311	9.88 ****	55.52 ****	1.64 **
	319	8.62 ****	34.42 ****	1.35 *

a. DOY, day of year – Julian calendar.
NS Not Significant at the < 0.05 level.
* Significant at the < 0.05 level.
** Significant at the < 0.01 level.
*** Significant at the < 0.001 level.
**** Significant at the < 0.0001 level.
