# Supplementary material for: Field-Based High-Throughput Plant Phenotyping Reveals the Temporal Patterns of Quantitative Trait Loci Associated with Stress-Responsive Traits in Cotton
Source: G3 (Bethesda). 2016 Jan 27;6(4):865–79. doi: 10.1534/g3.115.023515 (PMC4825657; doi:10.1534/g3.115.023515)
Supplement: Supporting Information [file supp_g3.115.023515_FigureS5.pdf]

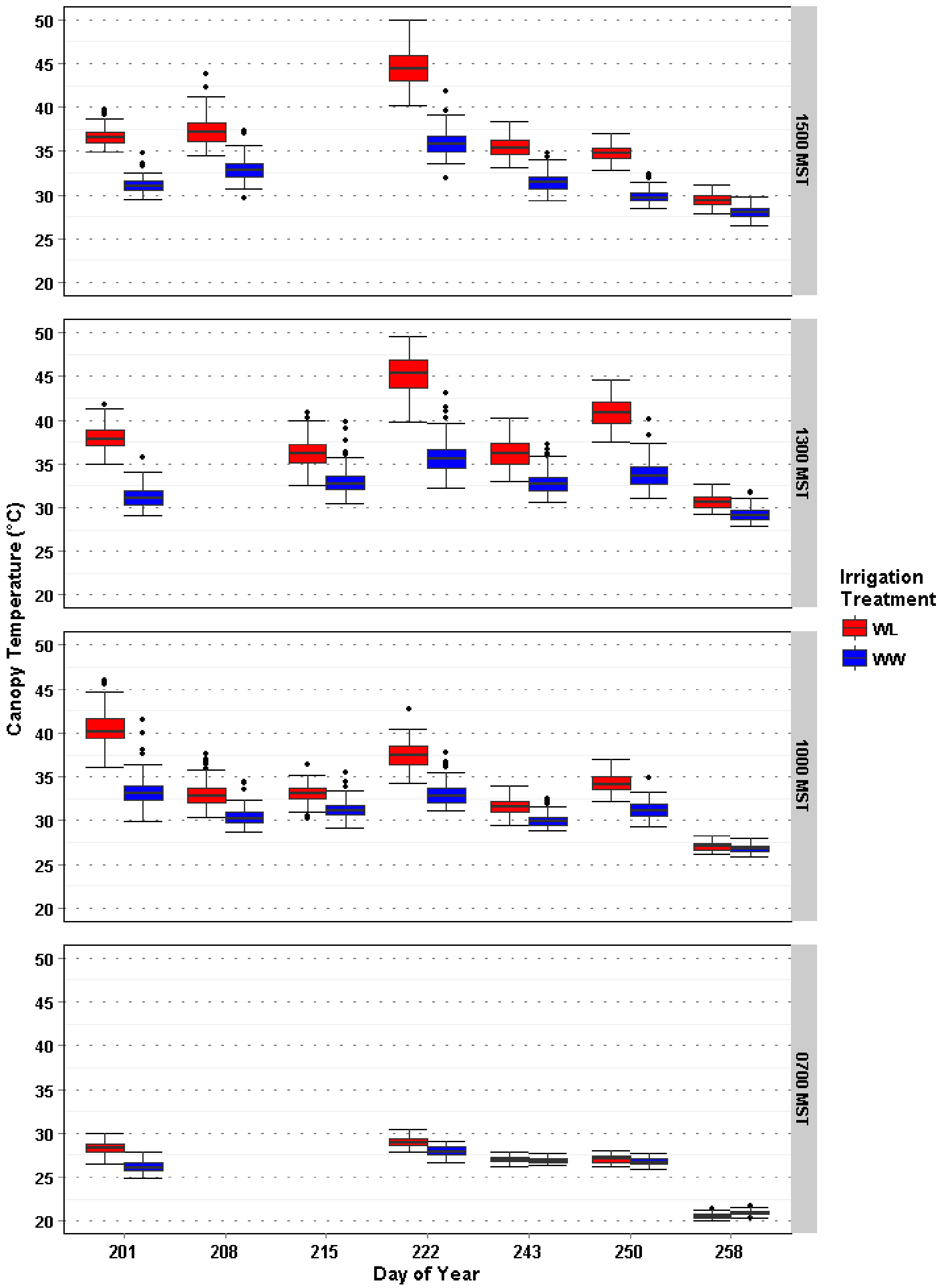

Figure S5 Box-and-whisker plots of best linear unbiased estimators (BLUEs) for canopy temperature (°C) collected from the TM-1×NM24016 mapping population and its parents for the 2012 growing season under two irrigation regimes, water-limited (WL) and well-watered (WW). The horizontal black line inside the box is the median.
