# Supplementary material for: Field-Based High-Throughput Plant Phenotyping Reveals the Temporal Patterns of Quantitative Trait Loci Associated with Stress-Responsive Traits in Cotton
Source: G3 (Bethesda). 2016 Jan 27;6(4):865–79. doi: 10.1534/g3.115.023515 (PMC4825657; doi:10.1534/g3.115.023515)
Supplement: Supporting Information [file supp_g3.115.023515_TableS24.pdf]

Table S24 Summary information for plant height in 2011. Plant height means, standard deviations, midparent values, and ranges of best linear unbiased estimators (BLUEs) for the TM-1×NM24106 recombinant inbred line (RIL) population and its two parents under two irrigation regimes, water-limited (WL) and well-watered (WW), in Maricopa, AZ in 2011.

**DOY**[a]	**Irrigation Regime**	**Parents**			**RIL population**			
		**TM-1**	**NM24016**	**Midparent**	**Mean**	**Std. Dev**	**Min.**	**Max.**
**189**	WL	0.52	0.49	0.51	0.49	0.05	0.41	0.59
	WW	0.56	0.53	0.54	0.51	0.04	0.41	0.60
**195**	WL	0.65	0.66	0.65	0.63	0.06	0.52	0.78
	WW	0.69	0.64	0.66	0.64	0.06	0.47	0.78
**203**	WL	0.71	0.64	0.67	0.68	0.07	0.56	0.86
	WW	0.72	0.77	0.75	0.72	0.07	0.55	0.93
**209**	WL	0.69	0.68	0.68	0.67	0.07	0.52	0.84
	WW	0.73	0.79	0.76	0.77	0.08	0.55	1.01
**216**	WL	0.72	0.67	0.70	0.73	0.08	0.56	1.01
	WW	0.75	0.80	0.78	0.80	0.08	0.62	1.03
**223**	WL	0.80	0.77	0.78	0.82	0.08	0.61	1.01
	WW	0.82	0.81	0.82	0.85	0.10	0.66	1.13
**231**	WL	0.69	0.75	0.72	0.79	0.10	0.58	1.07
	WW	0.74	0.83	0.79	0.84	0.12	0.58	1.25
**238**	WL	0.76	0.81	0.79	0.82	0.09	0.59	1.08
	WW	0.81	0.86	0.83	0.92	0.13	0.64	1.35
**244**	WL	0.86	0.89	0.87	0.89	0.10	0.66	1.27
	WW	0.85	0.83	0.84	0.91	0.14	0.60	1.33
**251**	WL	0.82	0.81	0.81	0.86	0.10	0.64	1.19
	WW	0.92	0.97	0.94	1.03	0.12	0.77	1.38
**299**	WL	0.87	0.85	0.86	0.92	0.11	0.71	1.27
	WW	0.94	0.89	0.92	1.01	0.14	0.72	1.44

a. DOY, day of year – Julian calendar.
