# Supplementary material for: Field-Based High-Throughput Plant Phenotyping Reveals the Temporal Patterns of Quantitative Trait Loci Associated with Stress-Responsive Traits in Cotton
Source: G3 (Bethesda). 2016 Jan 27;6(4):865–79. doi: 10.1534/g3.115.023515 (PMC4825657; doi:10.1534/g3.115.023515)
Supplement: Supporting Information [file supp_g3.115.023515_TableS25.pdf]

Table S25 Summary information for plant height in 2012. Plant height means, standard deviations, midparent values, and ranges of best linear unbiased estimators (BLUEs) for the TM-1×NM24106 recombinant inbred line (RIL) population and its two parents under two irrigation regimes, water-limited (WL) and well-watered (WW), in Maricopa, AZ in 2012.

DOY[a]	Irrigation Regime	Parents			RIL population			
		TM-1	NM24016	Midparent	Mean	Std. Dev	Min.	Max.
201	WL	0.65	0.62	0.64	0.64	0.06	0.45	0.80
	WW	0.68	0.62	0.65	0.65	0.07	0.52	0.80
209	WL	0.68	0.68	0.68	0.70	0.07	0.54	0.88
	WW	0.78	0.79	0.78	0.82	0.08	0.63	1.02
215	WL	0.66	0.68	0.67	0.74	0.10	0.50	1.02
	WW	0.80	0.91	0.85	0.91	0.10	0.66	1.15
223	WL	0.62	0.74	0.68	0.77	0.11	0.46	1.09
	WW	0.84	0.90	0.87	0.97	0.12	0.64	1.33
233	WL	0.64	0.69	0.67	0.79	0.12	0.52	1.15
	WW	0.83	0.91	0.87	0.99	0.13	0.69	1.39
240	WL	0.68	0.81	0.74	0.88	0.13	0.58	1.29
	WW	0.83	0.89	0.86	1.05	0.15	0.68	1.47
243	WL	0.69	0.82	0.76	0.88	0.12	0.59	1.21
	WW	0.83	0.87	0.85	0.98	0.14	0.64	1.42
250	WL	0.77	0.86	0.82	0.93	0.12	0.68	1.36
	WW	0.87	1.02	0.94	1.06	0.16	0.65	1.50
257	WL	0.78	0.86	0.82	0.90	0.12	0.56	1.36
	WW	0.86	0.90	0.88	1.03	0.17	0.66	1.63
311	WL	0.87	0.88	0.87	0.95	0.13	0.69	1.43
	WW	0.93	1.00	0.96	1.06	0.15	0.74	1.56
319	WL	0.89	0.96	0.93	0.99	0.12	0.74	1.47
	WW	0.94	0.90	0.92	1.08	0.16	0.77	1.60

a. DOY, day of year – Julian calendar.
