# Supplementary material for: Field-Based High-Throughput Plant Phenotyping Reveals the Temporal Patterns of Quantitative Trait Loci Associated with Stress-Responsive Traits in Cotton
Source: G3 (Bethesda). 2016 Jan 27;6(4):865–79. doi: 10.1534/g3.115.023515 (PMC4825657; doi:10.1534/g3.115.023515)
Supplement: Supporting Information [file supp_g3.115.023515_TableS26.pdf]

Table S26 Heritability estimates for plant height. Estimates of broad-sense heritability ($\hat{H}^2$) on an entry-mean basis for plant height for the TM-1×NM24106 recombinant inbred line (RIL) population evaluated under two irrigation regimes, water-limited (WL) and well-watered (WW) conditions.

Year	DOY[a]	Irrigation Regime	$\hat{H}^2$
2010	208	WL	0.77
		WW	0.74
	218	WL	0.77
		WW	0.74
	342	WL	0.83
		WW	0.85
2011	189	WL	0.58
		WW	0.73
	195	WL	0.67
		WW	0.73
	203	WL	0.61
		WW	0.68
	209	WL	0.75
		WW	0.64
	216	WL	0.64
		WW	0.72
	223	WL	0.67
		WW	0.84
	231	WL	0.79
		WW	0.79
	238	WL	0.75
		WW	0.84
	244	WL	0.75
		WW	0.83
	251	WL	0.77
		WW	0.74
	299	WL	0.63
		WW	0.83
2012	201	WL	0.48
		WW	0.65
	209	WL	0.72
		WW	0.71
	215	WL	0.77
		WW	0.75
	223	WL	0.74
		WW	0.86
	233	WL	0.86
		WW	0.85
	240	WL	0.82
		WW	0.84
	243	WL	0.83
		WW	0.87
	250	WL	0.84
		WW	0.86
	257	WL	0.73
		WW	0.89
	311	WL	0.82
		WW	0.86
	319	WL	0.79
		WW	0.84

a. DOY, day of year – Julian calendar.
