# Supplementary material for: Field-Based High-Throughput Plant Phenotyping Reveals the Temporal Patterns of Quantitative Trait Loci Associated with Stress-Responsive Traits in Cotton
Source: G3 (Bethesda). 2016 Jan 27;6(4):865–79. doi: 10.1534/g3.115.023515 (PMC4825657; doi:10.1534/g3.115.023515)
Supplement: Supporting Information [file supp_g3.115.023515_TableS27.pdf]

Table S27 Fixed effects for physiological traits within years. F values for fixed effects from an analysis of variance (ANOVA) for the TM-1×NM24106 recombinant inbred line (RIL) population, its two parents, and commercial check varieties for physiological traits for each individual year from 2010-12 at the Maricopa Agricultural Center located in Maricopa, AZ.

**Trait**	**Year**	**Source**		
		Genotype	Irrigation Regime	Genotype*Irrigation Regime
**ABA conc.**	2011	1.32*	10.86**	0.93 NS
	2012	1.50**	0.88 NS	1.36*
**Sugar conc.**	2011	1.40*	0.37 NS	1.07 NS
	2012	1.65**	11.93**	0.93 NS
**$\Delta^{13}C$**	2010	5.64****	1.04 NS	1.48**
	2011	3.40****	2.89 NS	1.35*
	2012	5.16****	40.80****	1.57**

NS Not Significant at the < 0.05 level.
* Significant at the < 0.05 level.
** Significant at the < 0.01 level.
*** Significant at the < 0.001 level.
**** Significant at the < 0.0001 level.
