# Supplementary material for: Field-Based High-Throughput Plant Phenotyping Reveals the Temporal Patterns of Quantitative Trait Loci Associated with Stress-Responsive Traits in Cotton
Source: G3 (Bethesda). 2016 Jan 27;6(4):865–79. doi: 10.1534/g3.115.023515 (PMC4825657; doi:10.1534/g3.115.023515)
Supplement: Supporting Information [file supp_g3.115.023515_TableS28.pdf]

Table S28 Summary of the non-linear relationship between canopy temperature and lint yield across cotton plant growth stages. The r^2 values are from fitted second order polynomial models of canopy temperature best linear unbiased estimators (BLUEs) regressed on lint yield BLUEs for years 2010-12 in the TM-1×NM24106 recombinant inbred line (RIL) population and its two parents under two irrigation regimes, water-limited (WL) and well-watered (WW), in Maricopa, AZ. All associations were significant at $\alpha = 0.05$, shown in bold.

Year	Cotton Plant Growth Stage	DOY[a]	TOD[b]	r^2 WL	r^2 WW
**2010**	Flowering/peak bloom	217	0700	**0.20**	0.06
		217	1300	**0.25**	**0.11**
	Boll development & fill	224	0700	**0.12**	0.01
		224	1000	**0.19**	**0.11**
		224	1300	**0.19**	0.03
		231	0700	**0.07**	0.03
		231	1000	0.05	0.04
		231	1300	**0.10**	0.02
**2011**	Flowering/peak bloom	188	0700	**0.39**	**0.28**
		188	1300	**0.34**	**0.22**
		195	0700	**0.24**	**0.19**
		195	1500	**0.30**	**0.18**
		202	0700	**0.17**	0.06
		202	1300	**0.13**	**0.15**
	Boll development & fill	216	0700	**0.09**	0.03
		216	1100	**0.09**	0.04
		216	1500	**0.09**	0.04
		223	0700	0.03	0.03
		223	1100	0.01	0.03
		223	1500	0.04	0.02
	Fiber development & elongation	230	0700	0.03	0.04
		230	1100	0.00	0.02
		230	1500	0.03	0.05
		237	0700	0.02	0.06
		237	1100	0.03	0.03
		237	1500	0.02	**0.07**
		244	0700	0.01	**0.08**
		244	1100	0.01	0.04
		244	1500	0.01	0.03
		251	0700	0.05	**0.12**
		251	1100	0.06	0.06
		251	1500	**0.15**	**0.10**
**2012**	Flowering/peak bloom	201	0700	**0.10**	**0.18**
		201	1000	**0.18**	**0.22**
		201	1300	**0.16**	**0.20**
		201	1500	**0.11**	**0.21**
	boll development & fill	208	1000	**0.18**	**0.24**
		208	1500	**0.12**	**0.23**
		215	1000	**0.08**	**0.12**
		215	1300	**0.12**	**0.16**
		222	0700	**0.07**	0.06
		222	1000	0.05	**0.15**
		222	1300	0.02	**0.08**
		222	1500	0.03	**0.11**
	Fiber development & elongation	243	0700	0.03	0.06
		243	1000	0.03	**0.08**
		243	1300	0.04	**0.10**
		243	1500	0.04	**0.08**
		250	0700	**0.09**	0.02
		250	1000	**0.08**	0.05
		250	1300	0.03	0.05
		250	1500	**0.09**	**0.10**

		258	0700	0.06	**0.08**
		258	1000	**0.07**	0.03
		258	1300	**0.08**	0.05
		258	1500	**0.06**	0.04

a. DOY, day of year – Julian calendar.

b. TOD, time of day within the day of year – MST.
