# Supplementary material for: Field-Based High-Throughput Plant Phenotyping Reveals the Temporal Patterns of Quantitative Trait Loci Associated with Stress-Responsive Traits in Cotton
Source: G3 (Bethesda). 2016 Jan 27;6(4):865–79. doi: 10.1534/g3.115.023515 (PMC4825657; doi:10.1534/g3.115.023515)
Supplement: Supporting Information [file supp_g3.115.023515_TableS29.pdf]

**Table S29 Summary of QTL for canopy temperature.** Summary of quantitative trait loci (QTL) identified from multi-environment, inclusive composite interval mapping (ME-ICIM) for canopy temperature in the TM-1×NM24016 recombinant inbred line (RIL) population under two irrigation regimes, water-limited (WL) and well-watered (WW), with an experiment-wise Type I error rate of 5%. Marker positions are reported as centimorgans (cM).

| Year[a] | Days[b] | Irrigation Regime[c] | Chr.[d] | LG[e] | Left marker | Left marker position (cM) | Right marker | Right marker position (cM) | Max LOD(A)[f] | Ave. LOD(A)[g] | Max PVE(A)[h] | Ave. PVE(A)[i] | Max additive effect[j] | Ave. additive effect[k] |
|---|---|---|---|---|---|---|---|---|---|---|---|---|---|---|
| **2** | 8 | WL/WW | A01 | 1 | DPL1673a | 33.71 | SNP0366 | 40.44 | 5.82 | 4.36 | 10.98 | 6.22 | -0.47 | -0.29 |
| **2** | 8 | WL/WW | A05 | 74 | SNP0029 | 0.00 | SNP0159 | 24.03 | 14.36 | 7.04 | 16.96 | 8.47 | 0.46 | 0.33 |
| **2** | 7 | WL/WW | A06 | 21 | SNP0146 | 9.23 | SNP0426 | 20.03 | 5.89 | 4.72 | 12.59 | 7.16 | -0.57 | -0.31 |
| **2** | 14 | WL/WW | A07 | 24 | DPL0790a | 0.00 | CIR238a | 16.60 | 9.29 | 5.50 | 13.77 | 8.51 | -0.47 | -0.33 |
| **3** | 12 | WL/WW | A08 | 29 | C2-003a | 0.00 | SNP0471 | 16.44 | 6.77 | 4.53 | 15.17 | 7.59 | -0.62 | -0.34 |
| **3** | 6 | WL/WW | A09 | 32 | SNP0093 | 20.32 | SNP0003 | 35.83 | 5.67 | 4.21 | 7.47 | 5.35 | 0.44 | 0.25 |
| **2** | 8 | WL/WW | A12 | 45 | SNP0264 | 0.00 | SNP0136 | 22.20 | 5.51 | 4.15 | 8.20 | 5.26 | 0.44 | 0.28 |
| **2** | 7 | WL/WW | A13 | 55 | MUSS181a | 37.24 | SNP0036 | 54.82 | 6.36 | 4.40 | 10.10 | 6.43 | 0.45 | 0.32 |
| **2** | 14 | WL/WW | D01 | 24 | DPL0790a | 0.00 | CIR238a | 16.60 | 9.29 | 5.50 | 13.77 | 8.51 | -0.47 | -0.33 |
| **2** | 3 | WL/WW | D01 | 65 | SNP0268 | 12.64 | SNP0465 | 24.70 | 4.10 | 3.77 | 4.67 | 4.35 | -0.26 | -0.24 |
| **2** | 9 | WL/WW | D04 | 96 | SHIN-1547 | 0.00 | SNP0285 | 15.80 | 13.00 | 6.45 | 14.67 | 7.98 | -0.51 | -0.33 |
| **2** | 4 | WW | D06 | 22 | SNP0044 | 6.75 | SNP0086 | 21.39 | 5.48 | 4.55 | 11.62 | 8.18 | -0.47 | -0.34 |
| **3** | 14 | WL/WW | D10 | 88 | DPL1903 | 0.00 | SNP0120 | 19.69 | 6.85 | 4.36 | 13.48 | 6.36 | 0.46 | 0.30 |
| **3** | 14 | WL/WW | D12 | 54 | SNP0331 | 0.00 | SNP0425 | 18.47 | 12.10 | 6.48 | 14.77 | 8.59 | -0.51 | -0.29 |

a. Year, number of years in which quantitative trait loci (QTL) were detected, years are 2010-2012.
b. Days, number of days across years on which data were collected, total of 19 unique days.
c. Irrigation regime, irrigation regime in which the QTL was identified.
d. Chr., chromosome on which marker is located.
e. LG, linkage group in which marker is located.
f. Max LOD(A), maximum logarithm of odds (LOD) for the QTL additive effect detected across all significant results for detected QTL.
g. Ave. LOD(A), average logarithm of odds (LOD) for the QTL additive effect averaged across all significant results for detected QTL.
h. Max PVE(A), maximum phenotypic variance explained by the additive effect of the detected QTL across all significant results, as a percentage.
i. Ave. PVE(A), average phenotypic variance explained by the additive effect of the detected QTL across all significant results, as a percentage.
j. Max additive effect, maximum additive effect when substituting a NM24016 allele with an allele from TM-1 across all significant results for detected QTL, reported as °C.
k. Ave. additive effect, average additive effect when substituting a NM24016 allele with an allele from TM-1, averaged across all significant results for detected QTL, reported as °C.
