# Supplementary material for: Field-Based High-Throughput Plant Phenotyping Reveals the Temporal Patterns of Quantitative Trait Loci Associated with Stress-Responsive Traits in Cotton
Source: G3 (Bethesda). 2016 Jan 27;6(4):865–79. doi: 10.1534/g3.115.023515 (PMC4825657; doi:10.1534/g3.115.023515)
Supplement: Supporting Information [file supp_g3.115.023515_TableS30.pdf]

Table S30 Summary of QTL for NDVI. Summary of quantitative trait loci (QTL) identified from multi-environment, inclusive composite interval mapping (ME-ICIM) for normalized difference vegetation index (NDVI) in the TM-1×NM24016 recombinant inbred line (RIL) population under two irrigation regimes, water-limited (WL) and well-watered (WW), with an experiment-wise Type I error rate of 5%. Marker positions are reported as centimorgans (cM).

Year[a]	Days[b]	Irrigation Regime[c]	Chr.[d]	LG[e]	Left marker	Left marker position (cM)	Right marker	Right marker position (cM)	Max LOD(A)[f]	Ave. LOD(A)[g]	Max PVE(A)[h]	Ave. PVE(A)[i]	Max additive effect[j]	Ave. additive effect[k]
3	10	WL/WW	A01	1	SNP0373	0.00	SNP0236	12.32	9.09	4.84	12.53	6.12	0.02	0.01
3	9	WL/WW	A03	6	SNP0001	4.25	DPL0170a	30.65	8.94	5.28	9.55	5.82	-0.02	-0.01
2	7	WL/WW	A03	71	SHIN-0690a	0.00	SHIN-0727a	10.86	6.05	4.30	5.85	4.92	-0.01	-0.01
3	12	WL/WW	A05	74	SNP0029	0.00	SNP0159	24.03	18.14	8.92	24.12	12.42	-0.03	-0.02
2	8	WL/WW	A06	21	SNP0146	9.23	SNP0426	20.03	6.99	4.68	11.10	6.97	0.02	0.02
3	19	WL/WW	A08	29	C2-003a	0.00	SNP0471	16.44	15.34	6.54	23.87	10.42	0.04	0.02
3	8	WL	A10	36	DPL0431a	27.12	TMB1288a	51.79	4.16	3.73	5.59	4.19	-0.01	-0.01
2	8	WL/WW	A11	91	SNP0476	0.00	SNP0227	22.70	6.14	4.86	16.69	10.15	0.02	0.02
2	13	WL/WW	A12	45	MUSB1117a	9.76	SHIN-1413a	33.25	8.98	5.19	11.85	6.87	-0.02	-0.01
2	11	WL/WW	A13	55	SNP0144	33.44	SNP0036	54.82	8.19	5.15	13.56	7.08	-0.02	-0.01
2	13	WL/WW	D01	24	DPL0790a	0.00	CIR238a	16.60	11.40	6.35	13.30	9.86	0.02	0.02
2	10	WL/WW	D04	96	SHIN-1547	0.00	SNP0285	15.80	7.70	4.82	11.17	6.69	0.02	0.01
2	12	WW	D06	22	SNP0132	0.00	SNP0028	18.90	7.31	5.24	11.22	7.11	0.02	0.01
3	15	WL/WW	D10	88	DPL1903	0.00	SNP0120	19.69	10.91	5.61	15.76	8.14	-0.03	-0.02
2	11	WW	D12	54	SNP0331	0.00	SNP0425	18.47	12.80	6.96	16.31	9.51	0.02	0.02

a. Year, number of years in which quantitative trait loci (QTL) were detected, years are 2010-2012.
b. Days, number of days across years on which data were collected, total of 20 unique days.
c. Irrigation regime, irrigation regime in which the QTL was identified.
d. Chr., chromosome on which marker is located.
e. LG, linkage group in which marker is located.
f. Max LOD(A), maximum logarithm of odds (LOD) for the QTL additive effect detected across all significant results for detected QTL.
g. Ave. LOD(A), average logarithm of odds (LOD) for the QTL additive effect averaged across all significant results for detected QTL.
h. Max PVE(A), maximum phenotypic variance explained by the additive effect of the detected QTL across all significant results, as a percentage.
i. Ave. PVE(A), average phenotypic variance explained by the additive effect of the detected QTL across all significant results, as a percentage.
j. Max additive effect, maximum additive effect when substituting a NM24016 allele with an allele from TM-1 across all significant results for detected QTL.
k. Ave. additive effect, average additive effect when substituting a NM24016 allele with an allele from TM-1, averaged across all significant results for detected QTL.
