# Supplementary material for: Field-Based High-Throughput Plant Phenotyping Reveals the Temporal Patterns of Quantitative Trait Loci Associated with Stress-Responsive Traits in Cotton
Source: G3 (Bethesda). 2016 Jan 27;6(4):865–79. doi: 10.1534/g3.115.023515 (PMC4825657; doi:10.1534/g3.115.023515)
Supplement: Supporting Information [file supp_g3.115.023515_TableS31.pdf]

Table S31 Summary of QTL for canopy height. Summary of quantitative trait loci (QTL) identified from multi-environment, inclusive composite interval mapping (ME-ICIM) for canopy height in the TM-1×NM24016 recombinant inbred line (RIL) population under two irrigation regimes, water-limited (WL) and well-watered (WW), with an experiment-wise Type I error rate of 5%. Marker positions are reported as centimorgans (cM).

Year[a]	Days[b]	Irrigation Regime[c]	Chr. [d]	LG[e]	Left marker	Left marker position (cM)	Right marker	Right marker position (cM)	Max LOD(A)[f]	Ave. LOD(A)[g]	Max PVE(A)[h]	Ave. PVE(A)[i]	Max additive effect[j]	Ave. additive effect[k]
2	10	WL/WW	A03	6	MUCS407a	0.00	SNP0084	10.76	10.92	6.38	12.95	8.98	0.05	-0.03
2	8	WL/WW	A03	71	SHIN-0690a	0.00	SHIN-0727a	10.86	5.57	4.25	9.78	6.25	-0.04	-0.03
2	9	WL/WW	A05	14	SNP0354	0.00	SHIN-0090a	10.23	6.88	4.70	9.12	6.33	-0.04	-0.03
2	11	WL/WW	A05	74	SNP0029	0.00	SNP0316	16.41	13.48	7.05	15.84	9.81	-0.04	-0.03
2	11	WL/WW	A06	17	SNP0191	14.08	SNP0369	23.23	7.12	4.77	16.38	8.64	0.04	0.03
2	11	WL/WW	A06	19	SNP0479	7.31	SNP0070	13.80	12.18	6.40	13.42	9.32	-0.04	-0.03
2	7	WL/WW	A10	36	DPL0431a	27.12	TMB1288a	51.79	4.94	4.23	7.89	5.67	-0.03	-0.03
2	13	WL/WW	A11	42	SNP0384	1.82	MUCS028	20.25	6.39	4.27	10.95	6.72	0.04	0.03
2	9	WL/WW	A11	91	SNP0430	18.09	DPL0253a	33.26	10.06	5.20	11.58	7.33	0.05	0.03
2	12	WL/WW	A12	45	MUSB1117a	9.76	SHIN-1413a	33.25	11.37	6.81	15.17	9.31	-0.05	-0.03
2	8	WL/WW	A12	47	DPL0010a	0.00	DPL1575a	16.06	9.14	5.35	11.89	7.01	-0.05	-0.03
2	10	WL/WW	D06	22	SNP0132	0.00	SNP0028	18.90	12.77	5.50	16.85	8.49	0.05	0.03
2	9	WW	D06	110	SNP0337	6.78	DPL0080a	12.38	6.43	5.16	14.23	8.58	-0.04	-0.03
2	9	WL/WW	D12	54	SNP0331	0.00	SNP0425	18.47	11.27	6.18	14.15	9.11	0.05	0.03

a. Year, number of years in which quantitative trait loci (QTL) were detected, years are 2011 and 2012.
b. Days, number of days across years on which data were collected, total of 14 unique days.
c. Irrigation regime, irrigation regime in which the QTL was identified.
d. Chr., chromosome on which marker is located.
e. LG, linkage group in which marker is located.
f. Max LOD(A), maximum logarithm of odds (LOD) for the QTL additive effect detected across all significant results for detected QTL.
g. Ave. LOD(A), average logarithm of odds (LOD) for the QTL additive effect averaged across all significant results for detected QTL.
h. Max PVE(A), maximum phenotypic variance explained by the additive effect of the detected QTL across all significant results, as a percentage.
i. Ave. PVE(A), average phenotypic variance explained by the additive effect of the detected QTL across all significant results, as a percentage.
j. Max additive effect, maximum additive effect when substituting a NM24016 allele with an allele from TM-1 across all significant results for detected QTL, reported as meters.
k. Ave. additive effect, average additive effect when substituting a NM24016 allele with an allele from TM-1, averaged across all significant results for detected QTL, reported as meters.
