# Supplementary material for: Field-Based High-Throughput Plant Phenotyping Reveals the Temporal Patterns of Quantitative Trait Loci Associated with Stress-Responsive Traits in Cotton
Source: G3 (Bethesda). 2016 Jan 27;6(4):865–79. doi: 10.1534/g3.115.023515 (PMC4825657; doi:10.1534/g3.115.023515)
Supplement: Supporting Information [file supp_g3.115.023515_TableS32.pdf]

Table S32 Summary of QTL for plant height. Summary of quantitative trait loci (QTL) identified from inclusive composite interval mapping (ICIM) for plant height in the TM-1×NM24016 recombinant inbred line (RIL) population under two irrigation regimes, water-limited (WL) and well-watered (WW), with an experiment-wise Type I error rate of 5%. Marker positions are reported as centimorgans (cM).

Year[a]	Days[b]	Irrigation Regime[c]	Chr.[d]	LG[e]	Left marker	Left marker position (cM)	Right marker	Right marker position (cM)	Max LOD(A)[f]	Ave. LOD(A)[g]	Max PVE(A)[h]	Ave. PVE(A)[i]	Max additive effect[j]	Ave. additive effect[k]
3	7	WL/WW	A06	19	DPL0080b	8.64	SNP0070	13.80	6.15	4.39	23.66	17.84	-0.06	-0.05
1	1	WL	A09	34	SNP0480	18.43	SNP0451	19.53	3.85	3.85	13.57	13.57	0.04	0.04
2	5	WL/WW	A11	42	DC30147a	0.00	MUCS028	20.25	6.35	4.31	32.51	16.81	0.06	0.04
1	1	WL	A12	45	DPL0248a	25.76	SHIN-1413a	33.25	3.59	3.59	12.42	12.42	-0.03	-0.03
1	1	WW	A12	47	DPL1575a	16.06	SNP0198	20.31	3.80	3.80	12.25	12.25	-0.04	-0.04
2	4	WW	D06	22	SNP0021	14.26	SNP0028	18.90	5.17	4.25	20.74	15.27	0.06	0.05
2	2	WW	D06	110	UCD311a	8.13	TMB0313a	16.13	3.47	3.47	16.91	14.85	-0.05	-0.04
1	2	WW	D10	88	DPL1022a	14.58	SNP0405	16.27	3.87	3.69	14.79	13.08	-0.03	-0.03
1	1	WW	D12	54	SNP0425	18.47	DC40080a	31.42	3.56	3.56	15.24	15.24	0.05	0.05

a. Year, number of years in which quantitative trait loci (QTL) were detected, years are 2011 and 2012.
b. Days, number of days across years on which data were collected, total of 25 unique days.
c. Irrigation regime, irrigation regime in which the QTL was identified.
d. Chr., chromosome on which marker is located.
e. LG, linkage group in which marker is located.
f. Max LOD(A), maximum logarithm of odds (LOD) for the QTL additive effect detected across all significant results for detected QTL.
g. Ave. LOD(A), average logarithm of odds (LOD) for the QTL additive effect averaged across all significant results for detected QTL.
h. Max PVE(A), maximum phenotypic variance explained by the additive effect of the detected QTL across all significant results, as a percentage.
i. Ave. PVE(A), average phenotypic variance explained by the additive effect of the detected QTL across all significant results, as a percentage.
j. Max additive effect, maximum additive effect when substituting a NM24016 allele with an allele from TM-1 across all significant results for detected QTL, reported as meters.
k. Ave. additive effect, average additive effect when substituting a NM24016 allele with an allele from TM-1, averaged across all significant results for detected QTL, reported as meters.
