# Supplementary material for: Field-Based High-Throughput Plant Phenotyping Reveals the Temporal Patterns of Quantitative Trait Loci Associated with Stress-Responsive Traits in Cotton
Source: G3 (Bethesda). 2016 Jan 27;6(4):865–79. doi: 10.1534/g3.115.023515 (PMC4825657; doi:10.1534/g3.115.023515)
Supplement: Supporting Information [file supp_g3.115.023515_TableS34.pdf]

Table S34 Summary of QTL for agronomic, fiber quality and physiological traits. Summary of inclusive composite interval mapping (ICIM) for physiological, fiber quality, and agronomic traits in the TM-1×NM24016 recombinant inbred line (RIL) population under two irrigation regimes, water-limited (WL) and well-watered (WW), with an experiment-wise Type I error rate of 5%. Marker positions are reported as centimorgans (cM).

Trait	Irrigation Regime[a]	Chr.[b]	LG[c]	Peak position	Left marker	Left Marker Position (cM)	Right marker	Right Marker Position (cM)	LOD[d]	PVE[e]	Additive effect[f]
ABA conc.	WL	A13	55	47	SNP0104	45.70	SHIN-1452a	48.00	3.73	20.43	-1.23
$\Delta^{13}C$	WL	A09	32	4	NAU2354a	3.84	SHIN-1542a	4.70	3.38	13.48	0.10
Fiber elongation	WL	A02	3	12	SHIN-0129b	11.53	DC40319b	12.18	3.50	11.09	0.25
	WL	A11	44	1	SNP0058	0.00	SNP0140	1.12	4.23	13.96	-0.29
	WW	A11	44	1	SNP0058	0.00	SNP0140	1.12	4.92	16.30	-0.32
Fiber uniformity	WL	A11	91	16	SNP0257	15.29	SNP0430	18.09	3.88	17.78	0.41
Fiber micronaire	WL	A11	91	29	BNL2805a	28.64	BNL0625a	29.18	3.65	11.53	0.19
	WL	D06	110	6	SNP0045	2.95	SNP0390	6.35	4.04	18.05	-0.24
	WW	D06	110	6	SNP0045	2.95	SNP0390	6.35	4.71	17.27	-0.23
	WL	D06	112	14	SNP0427	13.70	SNP0361	17.02	3.70	12.12	0.20
Boll size	WL	A05	74	21	SNP0315	16.41	SNP0159	24.03	4.05	13.76	0.22
	WW	A05	74	21	SNP0315	16.41	SNP0159	24.03	5.63	17.11	0.27
	WW	A12	41	1	DPL0252a	0.00	DPL1379a	1.57	4.50	12.77	0.36
	WL	D11	89	24	DPL0863b	21.18	DC30147b	24.42	3.84	12.35	-0.20
Lint yield	WW	A03	6	18	SNP0068	13.59	BNL0226a	19.22	7.04	14.76	67.34
	WL	A06	19	13	SNP0154	12.87	SNP0070	13.80	3.60	8.58	42.34
	WW	D01	24	16	SNP0193	9.75	CIR238a	16.60	5.87	11.12	58.41
	WL	D08	101	6	SNP0111	3.11	SNP0038	8.91	3.75	9.29	-41.44
	WW	D08	101	6	SNP0111	3.11	SNP0038	8.91	3.61	7.09	-46.38
	WW	D09	97	8	SNP0073	6.52	SNP0060	8.84	3.94	7.32	55.85
	WW	D09	99	0	SNP0259	0.00	C2-021a	2.32	4.37	7.86	48.69
	WL	D12	48	0	DPL0070a	0.00	DC30107a	0.97	6.90	17.85	-80.79
Seed per boll	WL	A11	89	25	DC30147b	24.42	BNL1034a	38.18	3.44	12.94	-1.00
	WW	D09	99	0	SNP0259	0.00	C2-021a	2.32	5.02	15.39	1.08

a. Irrigation regime, irrigation regime in which the QTL was identified.
b. Chr., chromosome on which marker is located.
c. LG, linkage group.
d. LOD, logarithm of odds (LOD) value at the position of peak likelihood of a quantitative trait locus.
e. PVE, Phenotypic variance explained by identified QTL reported, percentage.
f. Additive Effect, effect when substituting a NM24016 allele with an allele from TM-1.
